# Molecular hydrogen triggers TRPC4-TRPC4AP-dependent reversible calcium transients via extracellular influx

**DOI:** 10.7150/thno.124352

**Published:** 2026-02-26

**Authors:** Pengxiang Zhao, Han Li, Zisong Cai, Xujuan Zhang, Xiaohu Wen, Ziyi Liu, Shihao Jiang, Xue Jiang, Jiateng Wang, Zheng Dang, Mengyu Liu, Fei Xie, Xuemei Ma

**Affiliations:** College of Chemistry and Life Science, Beijing University of Technology, Beijing 100124, P. R. China.

**Keywords:** calcium ion, calcium transient, molecular hydrogen, TRPC4, TRPC4AP, gasotransmitter

## Abstract

**Rationale:**

Hydrogen gas (H_2_) produces pleiotropic therapeutic actions, but the exact molecular targets and ion-channel-based signaling cascades that underlie these benefits remain elusive. H_2_ may regulate calcium ion (Ca^2+^)-dependent processes, but the direct involvement of H_2_ in Ca^2+^ signaling and its underlying molecular mechanisms are unknown. We propose that H_2_ functions as a gaseous messenger that selectively opens a plasma-membrane Ca^2+^ channel to evoke Ca^2+^ transients ([Ca^2+^_i_]_t_) while avoiding cytotoxic overload, thereby offering a mechanism for its diverse biological effects.

**Methods:**

This study employed real-time calcium imaging and CRISPR-Cas9 gene editing, with live-cell imaging to monitor real-time calcium signal intensity in living cells. Two-photon *in vivo* imaging was applied to detect real-time Ca^2+^ signals in the brain and dorsal skin of C57BL/6 mice carrying adeno-associated virus-delivered calcium sensors. Live-cell F-actin staining and a wound healing (scratch) assay were used to assess the effects of H_2_ on cell motility. Protein-protein docking and molecular dynamics simulations were performed to analyze the interaction interface and binding forces between TRPC4 and TRPC4AP in three-dimensional space. Additionally, RNA sequencing was performed to validate downstream biological effects and transcriptional regulation triggered by H_2_.

**Results:**

H_2_ elicited rapid and reversible [Ca^2+^_i_]_t_ across multiple cell types in a Ca^2+^- and concentration-dependent manner, an effect that was absent in TRPC4⁻/⁻ or TRPC4AP⁻/⁻ cells. *In vivo* imaging in mice expressing a genetically encoded Ca²⁺ sensor showed that H_2_ inhalation elevated Ca^2+^ signals in the motor cortex (M1 region) and dorsal skin. Functionally, live-cell imaging and wound-healing assays confirmed that H_2_-induced Ca^2+^ transients enhanced cell motility. Mechanistically, protein docking revealed a dual-arginine cluster within the CIRB domain of TRPC4; its interaction with TRPC4AP was essential for H_2_-evoked Ca^2+^ influx. Mutating these arginines to alanine residues completely abolishing the response. H_2_ triggered proton efflux and increased intracellular pH. Molecular dynamics simulations indicated that altered pH modulates the binding force between TRPC4 Arg730/Arg731 and TRPC4AP. Transcriptomic analysis further demonstrated that H_2_ activates calcium-related channels and promotes cytoskeletal remodeling and cell migration.

**Conclusions:**

This study identifies H_2_ as a novel gaseous signaling molecule that can regulate Ca^2+^ channels via the TRPC4-TRPC4AP axis. The 730Arg-731Arg motif in TRPC4 serves as a critical H_2_-sensitive site, enabling dynamic calcium homeostasis without overload. These findings provide a mechanistic framework for developing gas-controlled H_2_ regenerative therapeutics.

## Introduction

H_2_ is increasingly recognized as a biologically active gas with potential therapeutic applications across a variety of medical fields, including ischemia-reperfusion injury, neurodegenerative diseases, and wound healing 1, 2. The extensive mechanisms of action of H_2_ have been widely studied, including its significant antioxidant and anti-inflammatory properties, as well as its ability to activate stem cells 3. These research findings provide strong support for the clinical application of H_2_. Despite extensive research, the mechanisms underlying the biological effects of H_2_ remain incompletely understood.

Current research hypotheses propose several potential pathways: H_2_ selectively scavenges reactive oxygen species such as hydroxyl radicals (·OH) and peroxynitrite (ONOO⁻), modulates the functions of various enzymes 4, 5, and regulates key signaling pathways such as Nrf-2 6, NF-κB 7, and MAPK 8. Although these hypotheses provide an initial framework for understanding the effects of H_2_, its specific molecular targets and detailed signaling mechanisms have not yet been fully elucidated, indicating a significant gap in our understanding of gasotransmitter biology.

Recent breakthrough studies by Zhao *et al.* have elucidated novel mechanisms underlying H_2_-mediated tissue repair 9. Their study found that H_2_ promotes wound healing by inducing the early proliferation of autologous epidermal stem cells (EpSCs) and collagen deposition. Additionally, gene set enrichment analysis (GSEA) revealed that H_2_ treatment activates Ca^2+^-dependent cell adhesion pathways. This suggests that H_2_ may initiate the repair process by regulating Ca^2+^ signaling, which determines stem cell fate and modulates the remodeling of the extracellular matrix (ECM). Notably, Ma *et al.* further demonstrated that H_2_ can modulate neuronal excitability, consistent with the observations by Zhao *et al.* on Ca^2+^-dependent adhesion pathways, collectively suggesting that ion flux regulation may serve as a unifying mechanism.

Gas signaling molecules often exhibit multitarget effects, and ion channels, as the molecular interface for signal transduction, have become the key executors mediating their biological effects 10. For example, carbon monoxide (CO) participates in various physiological and pathological processes by modulating targets such as calcium-activated potassium channels (regulating membrane potential and vascular tone), voltage-gated potassium channels (affecting neuronal excitability), and L-type calcium channels (maintaining calcium homeostasis) 11, 12. Mustafa *et al.* confirmed that the hydrogen sulfide (H_2_S) donor NaHS can activate ATP-sensitive potassium (KATP) channels in HEK293 cells by persulfidation of the Cys43 site on the Kir 6.1 subunit 13, 14. Nitric oxide (NO) can indirectly phosphorylate and inhibit TRPC1/3/6 by activating the soluble guanylate cyclase (sGC)-cGMP-PKG signaling pathway, thereby regulating vascular smooth muscle tone 15. Some studies have shown that H_2_S can regulate TRPC5/1 activity, suggesting that it may act as a gaseous sensor for TRPC channels 16. These studies have laid the foundation for the research on how gaseous signaling molecules regulate cellular calcium homeostasis through redox modification or second messenger systems. The Transient Receptor Potential (TRP) channel superfamily, with numerous members serving as established cellular redox sensors, exhibits functions that are intricately modulated by reactive oxygen species and gaseous signaling molecules 17. Against this backdrop, the physiological effects of the gaseous molecule H_2_ have increasingly attracted attention.

Ca^2+^ acts as a universal second messenger, mediating a variety of physiological processes through dynamic concentration gradients and complex regulatory mechanisms 18. The elevation of cytoplasmic Ca^2+^ concentration serves as a universal signaling pathway, transmitting signals from the cell surface to the interior and regulating key cellular functions 19, including gene expression 20, cell cycle progression 21, motility 22, autophagy 23, and apoptosis 24. A conventional calcium transient is the dynamic process in which the Ca^2+^ concentration in the cell rapidly rises and then returns to baseline within a short period 25. In cardiomyocytes, conventional calcium transients are directly linked to key physiological processes, including myocardial contraction, relaxation, and excitation-contraction coupling 26, 27. In the nervous system, Ca^2+^ is crucial for neurotransmitter release 28, 29. The action potential of neurons triggers a presynaptic calcium transient, that neurotransmitters release, thereby facilitating communication between neurons. In addition, extracellular Ca^2+^ maintains the resting membrane potential in excitable cells, such as neurons and muscle cells 30, 31, and supports normal bone formation 32, 33.

Calcium agonists are compounds that increase the influx of Ca^2+^ through calcium channels in excitable tissues, which is essential for functions such as muscle contraction, vascular regulation, and hormone release 34, 35. However, there are significantly fewer identified calcium channel agonists compared to the numerous known calcium channel antagonists 36, 37.

This study employs a multidimensional experimental approach to explore whether ion channels can serve as targets for H_2_. Using live-cell Ca^2+^ imaging, *in vivo* two-photon imaging in small animals, gene-editing amino acid mutation technologies, transcriptomic analysis, and molecular docking, our research revealed that H_2_ induces a novel calcium transient [Ca^2+^_i_]_t_ via the TRPC4-TRPC4AP signaling axis, exerting effects similar to calcium agonists. These findings identify H_2_ as a novel gaseous signaling molecule that regulates Ca^2+^ channels. Our study aims to deepen our understanding of H_2_ signaling mechanisms, thereby paving the way for innovative therapeutic interventions targeting calcium signaling pathways.

## Results

### H_2_ triggers reversible calcium transients in cells

**Figure 1:**
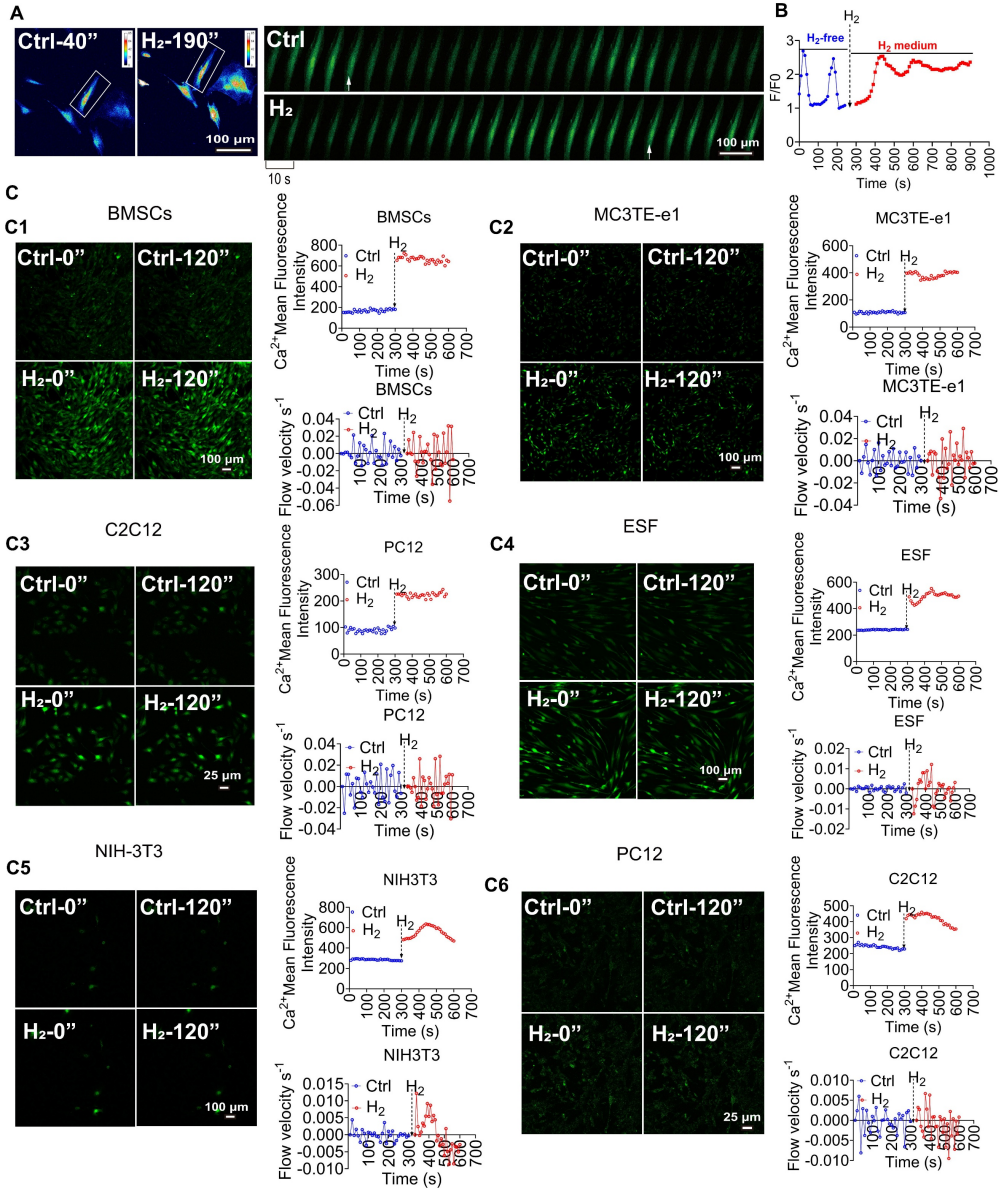
** [Ca^2+^_i_]_t_ induced by H_2_ is broad-spectrum. A.** Pseudocolor and time series images of [Ca^2+^_i_] changes in H_2_-free and H_2_-medium in MSCs. **B.** Fluo 4 averaged F/F0 trace in imaging H_2_-free (blue) and H_2_-medium (red). **C.** H_2_ stimulates calcium transients in various cells, including mouse bone marrow-derived mesenchymal stem cells (BMSCs,C1), mouse osteoblasts (MC3T3-E1,C2), mouse myoblasts (C2C12,C3), human skin fibroblasts (ESF,C4), mouse fibroblasts (NIH-3T3,C5), , and rat neuronal cells (PC12,C6); left panels show random field fluorescence images of Ca^2+^ at 0 and 120 s, and the right panels display the relative fluorescence intensity statistics and Ca^2+^ flow velocity measurements over 600 s, comparing the periods before and after the introduction of H_2_. Scale bar in A, C1, C2, C4, and C5 = 100 μm; Scale bar in C3 and C6 = 25 μm.

H_2_ can induce [Ca^2+^_i_] in cells. Previous research by Zhao *et al.* 9. found that H_2_ can promote the proliferation, differentiation, and motility of various skin stem cells. To verify whether H_2_ affects intracellular Ca^2+^ flux, we used human umbilical cord-derived mesenchymal stem cells (MSCs) as an experimental model. Our study found that under conventional culture conditions, cells exhibited periodic calcium oscillations (F/F_0_ peak). Exposure to saturated H_2_ (1.6 mg/L) 38 rapidly increased cytoplasmic Ca^2+^concentration (Figure 1A), reaching a peak within 30 s (F/F_0_ = 2.8 ± 0.3) (Figure 1B). Moreover, the Ca^2+^ signal remained stable in MSCs for over 30 minutes after H_2_ stimulation. When the signal intensity returned to baseline upon H_2_ removal, this response was reversible (Figure S1A), with spontaneous Ca^2+^ oscillations reappearing approximately 50 min after the peak of the high calcium signal, characterized by periodic calcium fluctuations, with a F/F_0_ peak of 1.2 ± 0.2 (Figure S2A).

To confirm the relationship between intracellular Ca^2+^ levels and H_2_, MSCs were treated with different concentrations of H_2_. We found that when the H_2_ concentration was reduced from the saturated concentration (1.6 mg/L) to half-saturation (0.8 mg/L), the Ca^2+^ fluorescence intensity decreased by 29.1% ± 12.4% ((Figure S2B). When the H_2_ concentration was further reduced to 0.4 mg/L, the Ca^2+^ fluorescence peak F/F_0_ rapidly declined (F/F_0_ = 1.3 ± 0.12), indicating that changes in intracellular Ca^2+^ levels were H_2_ dose-dependent. Notably, treating MSCs and HUVECs with H_2_ induced [Ca^2+^_i_] (Figure S3A-B). Upon removal of H_2_ at any time, the cell calcium signal intensity immediately returned to baseline levels (F/F₀ = 1.43 ± 0.58 for MSCs; F/F₀ = 1.24 ± 0.02 for HUVECs) and resumed periodic calcium oscillations, confirming that H_2_ was the cause of the intracellular Ca^2+^ changes. Crucially, this regulatory mechanism operated independently of oxygen tension (Figure S1B). We refer to this H_2_-dependent reversible increase in intracellular Ca^2+^ as a novel calcium transient [Ca^2+^_i_]_t_.

The H_2_-induced novel [Ca^2+^_i_]_t_ process in cells comprised two stages. In the first stage, during the normal periodic calcium oscillations of the cells, H_2_ was introduced, opening the calcium channels. The cytoplasmic Ca^2+^ concentration increasesd rapidly within 30 s, showing H_2_ dependency. We refer to this stage as the [Ca^2+^_i_] phase. In the second stage, as the local H_2_ concentration decreased, the intracellular Ca^2+^ concentration decreased gradually. Due to H_2_-induced opening of calcium channels, cells transiently elevated conventional calcium oscillations to maintain calcium homeostasis.

To determine whether H_2_-induced [Ca^2+^_i_]_t_ are universal, seven different cell models were systematically analyzed (Figure 1C), for changes in Ca^2+^ fluorescence intensity and Ca^2+^ flow velocity before and after H_2_. The results showed that bone marrow mesenchymal stem cells (BMSCs) (Figure 1C1), mouse osteoblasts (MC3T3E-e1) (Figure 1C2), mouse myoblasts (C2C12) (Figure 1C3), human skin fibroblasts (ESF) (Figure 1C4), mouse fibroblasts (NIH-3T3) (Figure 1C5), rat neuroblastoma cells (PC12) (Figure 1C6), all exhibited varying degrees of intracellular [Ca^2+^_i_]_t_ under H_2_ exposure.

### Extracellular [Ca^2+^_i_] is the primary source of [Ca^2+^_i_]_t_ induced by H_2_

**Figure 2:**
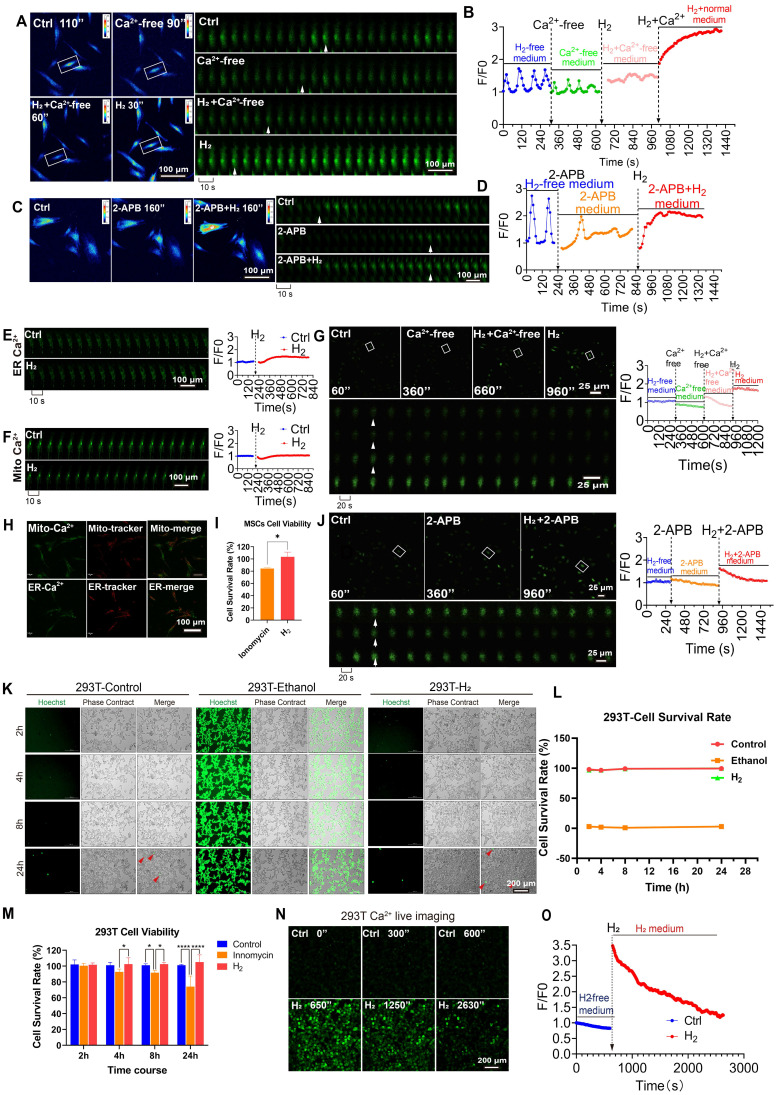
** H_2_ promotes [Ca^2+^_i_] without altering cellular activity or Ca^2+^ overload. A.** & **B**. Pseudocolor (A left) and time series (A right) images and the Fluo4 averaged F/F0 trace of the [Ca^2+^_i_] changes under H_2_-free, Ca^2+^-medium, H_2_+Ca^2+^free-medium, and then back to H_2_-medium in MSCs. **C & D**. Pseudocolor (C left) and time series (C right) images and the Fluo4 averaged F/F0 trace of the [Ca^2+^_i_] changes under H_2_-free, 2-APB-medium, and then back to 2-APB+H_2_-medium in MSCs.** E & F**. Time series images and the averaged F/F0 trace of the [Ca^2+^_i_] changes in ER (E) and Mito (F) under H_2_-free and H_2_-medium in MSCs. **H**.Co-localization of Mito-Ca^2+^ and ER-Ca^2+^ with their trackers. **G**.Time-lapse images and the Fluo4 averaged F/F0 trace of the [Ca^2+^_i_] changes under H_2_-free, Ca^2+^free-medium, H_2_+ Ca^2+^free-medium, and then back to H_2_-medium conditions in HUVEC.** J**. Time-lapse images and the Fluo4 averaged F/F0 trace of the [Ca^2+^_i_] changes in H_2_-free, 2-APB-medium, and then back to 2-APB+ H_2_-medium in HUVEC. **I & M**. The cell viability of MSCs and 293T remained unaffected by H_2_ exposure, in contrast to the significant cytotoxicity induced by the Ca^2+^ ionophore lonomycin. **K & L**. Live cell imaging of 293T cell death staining following 24 h of H_2_ treatment and survival rate, the ethanol-treated group served as the dead cell dye control. **N & O**. Time-lapse live cell imaging of H_2_-promoted [Ca^2+^_i_] dynamics and F/F0 fluorescence intensity analysis. A, C, E and F indicate cells at distinct time points within the time-lapse capture, sampling rate, 10 sec. G and J indicate cells at distinct time points within the time-lapse capture, sampling rate, 20 sec. Data in I and M were processed using an unpaired T test, and were plotted as Mean ± SEM. *P value < 0.05; **P value < 0.01; ***P value < 0.001; no stars for P value > 0.05. Scale bar in **A, C, E, F,** and **H** = 100 μm; Scale bar in G and J= 25 μm. Scale bar in K and N= 200 μm.

The accumulation of Ca^2+^ in the cytoplasm mainly originates from three sources: influx from the extracellular space 39, release from the endoplasmic reticulum Ca^2+^ stores 40, or release from mitochondrial Ca^2+^ 41. We introduced a Ca^2+^-free culture system in MSCs to determine the role of extracellular Ca^2+^ in H_2_-induced calcium signaling. We found that under Ca^2+^-free culture conditions (Figure 2A), H_2_ had no significant effect on cytoplasmic Ca^2+^, with calcium oscillations at baseline levels (F/F₀ = 1.08 ± 0.11) (Figure 2B). When the culture was switched to a Ca^2+^-containing, H_2_-saturated system, the Ca^2+^ signal rapidly reached an F/F₀ peak, and H_2_ restored the ability of MSCs to accumulate Ca^2+^ in the cytoplasm, with F/F₀ = 2.54 ± 0.27 (Figure 2B).

Similar observations were made in HUVECs. Under Ca^2+^-free culture conditions, H_2_ had no significant effect on cytoplasmic Ca^2+^. However, when the culture was switched to a Ca^2+^-containing, H_2_-saturated system, the Ca^2+^ signal rapidly reached an F/F₀ peak, and H_2_ restored the ability of HUVECs to accumulate Ca^2+^ in the cytoplasm (Figure 2G). These results indicated that extracellular Ca^2+^ is a crucial factor in the process of H_2_-induced intracellular [Ca^2+^_i_]_t_.

To determine the contribution of Ca^2+^ from the endoplasmic reticulum (ER) Ca^2+^ stores in H_2_-induced [Ca^2+^_i_]_t_, we used 2-aminoethoxydiphenyl borate (2-APB) to deplete cellular Ca^2+^ pharmacologically 42. Under conditions of specific endoplasmic reticulum Ca^2+^ (ER-Ca^2+^) store depletion, H_2_ still significantly increased the cytoplasmic Ca^2+^ concentration in MSCs (Figure 2C-D) and HUVECs (Figure 2J). Using fluorescence probes specific to ER-Ca^2+^ and mitochondrial Ca^2+^(Mito-Ca^2+^) dual-channel ratiometric fluorescence imaging was performed to quantify Ca^2+^ levels. The results showed no significant changes in Ca^2+^ levels within the ER or mitochondria under H_2_ exposure (Figure 2E-F). Additionally, co-localization studies of Mito-Ca^2+^ and ER-Ca^2+^ with their respective Ca^2+^ indicators revealed no significant changes in Ca^2+^ levels (Figure 2H). Statistical analysis based on fluorescence intensity indicated that the observed increase in intracellular Ca^2+^ signaling in MSCs was primarily due to extracellular [Ca^2+^_i_], with minimal contribution from ER-Ca^2+^ release and no contribution from mitochondria. These findings confirmed that extracellular [Ca^2+^_i_] plays a dominant role in H_2_-induced intracellular [Ca^2+^_i_]_t_ and the regulation of calcium homeostasis.

High intracellular Ca^2+^ can lead to severe cytotoxicity, with calcium overload as an intermediate state that disrupts oxidative phosphorylation and is associated with multi-organ dysfunction. We used ionomycin as a positive control for calcium overload and performed CCK8 assays. The results showed that after 24 of H_2_ treatment, MSCs had calcium levels higher than those in the ionomycin group, indicating that H_2_ treatment did not induce calcium (Figure 2I). We also validated this observation in the 293T cell line. Live/dead cell staining experiments revealed that cell viability was unaffected in the H_2_ group compared to the control (Figure 2K, L). At 4, 8, and 24 h, the viability of 293T cells was higher than that of the ionomycin group and did not differ significantly from that of the control group (Figure 2M). The H_2_-dependent reversible increase in intracellular Ca^2+^ [Ca^2+^_i_]_t_, could affect [Ca^2+^_i_] as the H_2_ concentration in the culture medium decreased. After a single H_2_ treatment of 293T cells, the intracellular Ca^2+^ concentration returned to levels comparable to the control group within 26-30 s (Figure 2N-O), indicating that H_2_-induced [Ca^2+^_i_]_t_ do not affect cell viability and are broadly applicable.

### H_2_ induces [Ca^2+^_i_] through the TRPC4-TRPC4AP axis, triggering [Ca^2+^_i_]_t_

**Figure 3:**
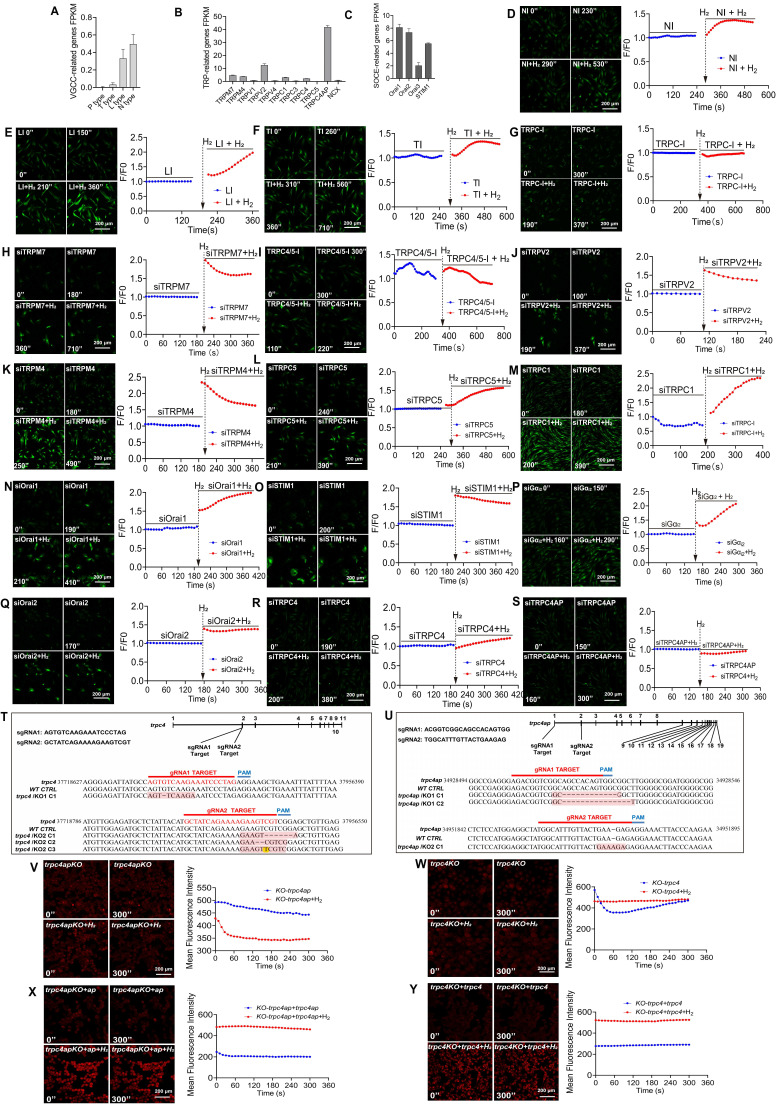
** H_2_ induces calcium ion influx via the TRPC4-TRPC4AP axis, triggering calcium transients in MSCs. A-C.** Bulk RNAseq analysis of MSCs reveals a comparative assessment of FPKM values for various types of Ca^2+^ channels and their associated gene expression. **D**-**G** &** I**. Screening for Ca^2+^ channels potentially targeted by H_2_, including N-, L-, T-type, TRPC, and TRPC4/5, using different inhibitors. **H** & **J-S.** Screening for the potential influences of siRNA-mediated TRPM7, TRPV2, TRPM4, TRPC5, TRPC1, Orai1, STIM1, Gα_i2_, Orai2, TRPC4 and TRPC4AP knockdown in [Ca^2+^_i_] triggered by H_2_. **T** & **U.** Confirmation of trpc4 and trpc4ap knockout in monoclonal 293T cell lines generated by CRISPR-Cas 9. For each sgRNA, the target sequence is shown. The mutant site is highlighted in red, and the protospacer adjacent motif (PAM) is underlined. Sequences of knockout cells were determined by next-generation sequencing. **V** & **W.** [Ca^2+^_i_] induced by H_2_ vanished following the knockout of trpc4 or trpc4 ap. graphs showing the Fluo4 averaged F/F0 trace of the [Ca^2+^_i_] changes under different conditions. **X** & **Y**. [Ca^2+^_i_] induced by H_2_ recovered after trpc4 or trpc4ap overexpression in the trpc4 or trpc4ap knockout cell lines; graphs showing the Fluo4 averaged F/F0 trace of the [Ca^2+^_i_] changes under different conditions. Figures A, B, and C display the FPKM values of genes associated with VGCC, TRP, and SOCE channels in MSC cells based on transcriptomic sequencing results. Relevant supporting materials are provided in the Supplementary Material. Scale bar = 200 μm.

Ca^2+^ channels can be classified into various types based on their structural and functional characteristics. To identify the calcium channels through which extracellular Ca^2+^ enters cells, we analyzed the FPKM values of calcium channel-related genes on the membrane of MSCs using transcriptome sequencing. We found relatively low expression levels of T-type 43, L-type 44, and N-type voltage-gated calcium channels (VGCCs) (Figure 3A). Among the transient receptor potential (TRP) channels, TRPM4, TRPM7, TRPV2, TRPC1 45, and TRPC4 showed significant expression (Figure 3B), with TRPC4AP as the most highly expressed gene in the TRP family. In the components of store-operated calcium entry (SOCE), Orai1, Orai2, and STIM1 were identified as the major expressed genes (Figure 3C) 46.

In MSCs, we conducted inhibitor and siRNA interference experiments targeting the identified calcium channel-related genes. Pharmacological inhibition of T-type, L-type, or N-type VGCCs had little effect on H_2_-enhanced [Ca^2+^_i_] (Figures 3D-F). Additionally, the H_2_-mediated effects were not affected by inhibitors of TRPM7, TRPV2, TRPM4, Orai1, STIM1, or Orai2 (Figures 3H, J, K, N, O, and Q). Notably, a broad TRPC family inhibitor (Figure 3G) and a specific TRPC4/5 antagonist (Figure 3I) effectively attenuated H_2_-induced [Ca^2+^_i_]. Importantly, H_2_-triggered [Ca^2+^_i_] was not affected by knockdown of Giα, confirming that its mechanism is independent of Giα signaling (Figure 3P).

TRPC1, TRPC4, and TRPC5 channel proteins often assemble into heterotetrameric complexes, and TRPC4 shares high homology with TRPC5. Gene silencing experiments indicated that H_2_-mediated [Ca^2+^_i_] does not depend on TRPC5 or TRPC1 (Figures 3L-M), but TRPC4 silencing significantly reduced [Ca^2+^_i_] in H_2_-induced [Ca^2+^_i_]_t_ (Figure 3R). Transient Receptor Potential Channel 4-Associated Protein (TRPC4AP) directly interacts with TRPC4. Knockdown of TRPC4AP completely abolished the regulation of H_2_ on [Ca^2+^_i_] (Figure 3S), indicating that TRPC4AP is a key molecule in H_2_-induced [Ca^2+^_i_]_t_.

We used CRISPR-Cas9 technology to establish TRPC4 and TRPC4AP gene-knockout 293T cell lines and elucidate the mechanisms of TRPC4 and TRPC4AP in H_2_ signaling (Figures 3T-U). Sequencing data confirmed the efficiency of the knockout (Figure S5), and TRPC4 (293Ttrpc4^-^) and TRPC4AP (293Ttrpc4ap^-^) cells completely lost their response to H_2_-induced [Ca^2+^_i_], with no [Ca^2+^_i_]_t_ response (Figures 3V-W). Reintroduction of wild-type TRPC4 (wt-trpc4) or TRPC4AP (wt-trpc4ap) plasmids restored the H_2_-induced [Ca^2+^_i_]_t_ response in TRPC4 (293Ttrpc4^-^) and TRPC4AP (293Ttrpc4ap^-^) cells (Figures 3X-Y). These findings provided definitive evidence for the crucial roles of TRPC4 and TRPC4AP in mediating H_2_-dependent [Ca^2+^_i_]_t_ signaling.

### Molecular docking prediction and validation of the TRPC4-TRPC4AP protein binding site

**Figure 4:**
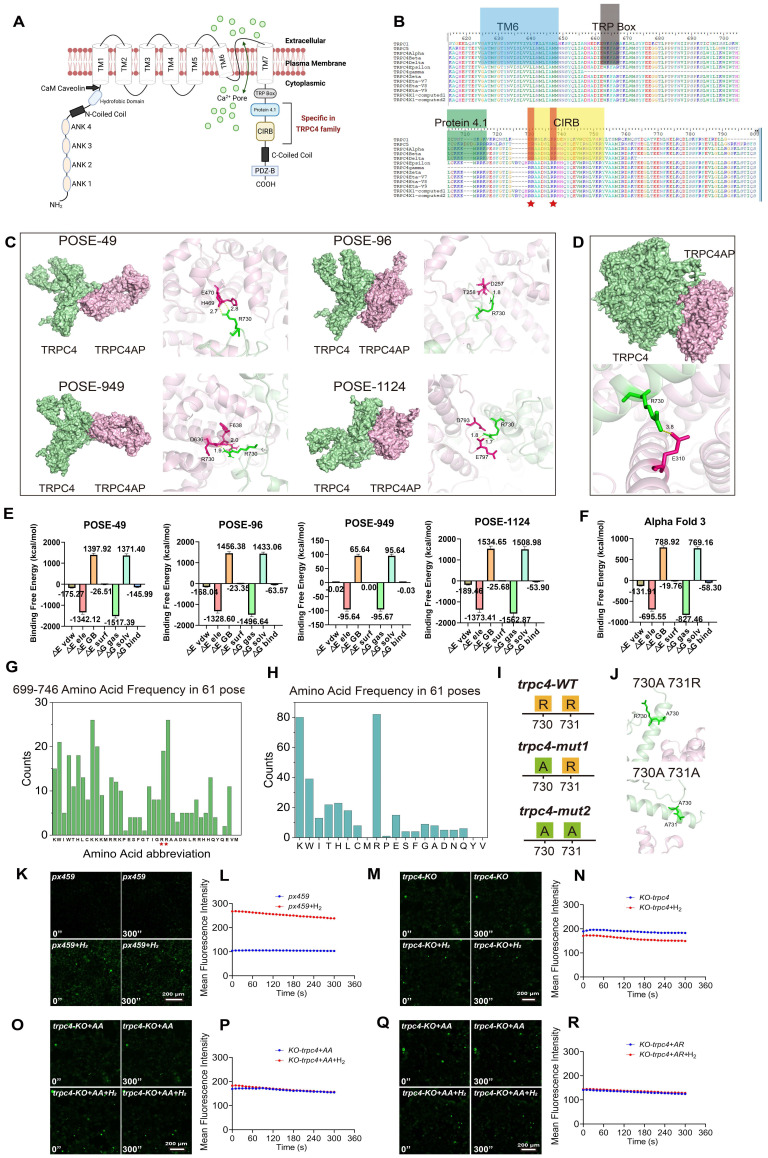
** Molecular docking prediction and validation of the binding site of TRPC4-TRPC4AP protein. A.** Diagram of the structure of a TRPC4 Ca^2+^ channel including specific motifs for the TRPC4 family. **B.** Amino acid sequence alignment of TRPC1, TRPC5, and 11 subtypes of the TRPC4 family within the TM6, TRP Box, Protein 4.1, and CIRB domains. The specific arginine residues within the TRPC4 family are highlighted, and red pentagrams indicate the areas of interest (arginine residues) in the CIRB motif in the sequence alignment. **C** & **D.** Molecular docking utilizing advanced protein-docking software (C) and the AlphaFold 3 platform (D) confirmed the interaction binding sites of TRPC4 and TRPC4ap, with consistent prediction outcomes. **E** & **F.** Free energy of the docking system.** G&H.** Amino acid frequency analysis at various positions (G) and total count (H) from the top 61 TRPC4-TRPC4ap combination patterns with the highest scores. Double Arginine residues are indicated with red pentagram symbol. **I.** Schematic representation of the wild-type and two mutant forms of the TRPC4 protein at positions 730 and 731 at the C-terminus. **J.** Molecular docking utilizing the AlphaFold 3 predicted the binding affinity of two TRPC4 mutants with TRPC4ap at positions 730 and 731, indicating a loss of binding interaction. **K**-**R.** [Ca^2+^_i_] induced by H_2_ and the Fluo4 averaged F/F0 trace of the [Ca^2+^_i_] changes under different conditions, including blank vehicle, trpc4-KO, trpc4-KO+Arg^730^Arg^731^, trpc4-KO+Ala^730^Arg^731^. (For E: △E vdw: van der Waals interaction energy; △E ele: electrostatic interaction energy; △E GB: polar solvation free energy; △E surf: nonpolar solvation free energy; △G gas: gas-phase binding free energy; △G solv: total solvation free energy; △G bind: calculated binding free energy.) Scale bar in K, M, O and Q= 200 μm.

We investigated the interaction between TRPC4 and TRPC4AP by protein-protein docking analysis using the structural file of TRPC4 (PDB ID:7b0j) and the TRPC4AP model predicted by AlphaFold 3 47. From 2000 potential docking conformations, we selected the top 60 TRPC4-TRPC4AP interaction modes based on computational scoring criteria 48, 49. Structural alignment revealed a unique cytokine-induced SH2 domain-binding (CIRB) domain in TRPC4 (Figure 4A-B), which is a key regulatory element in cellular signaling 50, 51. This domain typically facilitates interactions with SH2 domains to coordinate fundamental cellular processes, including growth regulation, differentiation programming, migration control, and survival mechanisms 52.

Detailed analysis of TRPC4-TRPC4AP complex formation indicated that the CIRB domain of TRPC4 mediates the interaction with TRPC4AP. By mapping the amino acid frequency of conserved regions in TRPC4 (Figure 4G-H) and combining computational modeling, we identified two adjacent arginine residues (positions 730 and 731) in the C-terminal region of the CIRB domain as the primary interaction sites 53. The corresponding structural models displayed the four highest-scoring binding modes (Figure 4C). Parallel prediction analysis using AlphaFold 3 and the same docking software showed consistent binding patterns, with interaction between TRPC4 arginine-730 and a glutamate residue on TRPC4AP achieving the highest Pairwise Shape Complementarity (PSC) value (Figure 4D).

To accurately evaluate the binding affinity, we calculated binding-free energy using the Molecular Mechanics Poisson-Boltzmann Surface Area (MMPBSA) method based on molecular dynamics (MD) trajectories 54, on four representative docking poses and the complex structure predicted by AlphaFold 3. The results revealed that the binding-free energy of the pose49 conformation (-145.99 kcal/mol) was significantly lower than those of the other docking poses (pose96: -63.57 kcal/mol; pose949: -0.03 kcal/mol; pose1124: -53.90 kcal/mol) (Figure 4E) and the AlphaFold 3 prediction (-58.30 kcal/mol) (Figure 4F). This set of quantitative thermodynamic data consistently indicated that pose49 was the most energetically favorable binding conformation, providing strong thermodynamic validation of our previous docking-based screening.

We conducted a systematic mutational analysis of arginine residues at positions 730 and 731 within the CIRB motif to elucidate the molecular basis of the TRPC4-TRPC4AP interaction. Structural predictions based on AlphaFold 3 showed that single (R730A) or double mutations (R730A/R731A) completely disrupted protein-protein interactions (Figure 4I-J), thereby establishing the crucial role of these conserved residues in complex formation.

To validate the molecular docking predictions, we constructed amino acid mutation vectors based on human amino acid preferences in the px459 system and generated a TRPC4 knockout cell line, trpc4KO. We mutated the wild-type arginines (wt730Arg731Arg) in the CIRB motif to 730Ala731Arg and 730Ala731Ala to study the H_2_ dependence on the TRPC4-TRPC4AP binding site. The results showed that in the presence of H_2_, Ca^2+^ concentration doubled in the px459 selection control group (Figure 4K-L), while the trpc4KO cell line did not respond to H_2_-induced [Ca^2+^_i_] (Figure 4M-N). After reconstituting the trpc4KO cell line with px459-730Ala730Ala (Figure 4O-P) and px459-730Ala731Arg (Figure 4Q-R), the enhancement of H_2_-mediated [Ca^2+^_i_] was lost. These findings confirmed that H_2_ enhances intracellular [Ca^2+^_i_] and the binding site of TRPC4-TRPC4AP, composed of 730Arg731Arg in the CIRB motif, is crucial for Ca^2+^ channel opening.

These experimental results underscore that interactions between acidic and basic amino acids can form spatially compatible and energetically favorable binding features. To evaluate the environmental sensitivity of this binding mechanism, we further investigated the effect of physiological pH conditions on the key basic residues (Arg730 and Arg731). Using PROPKA3 and CHARMM-GUI, we predicted and constructed protonation state models at different pH values (7.4 vs. 8.0), followed by MD simulations and inter-residual interaction energy calculations.

The results indicated that in the pose 49 conformation, Arg730 and Arg731 exhibited the strongest total interaction force (predominantly electrostatic) with the protein TRPC4AP. When the pH increased from 7.4 to 8.0, this total interaction weakened from -455.68 kJ/mol (Arg730=-209.48 kJ/mol; Arg731=-246.20 kJ/mol) to -429.37 kJ/mol (Arg730=-201.83 kJ/mol; Arg731=-227.54 kJ/mol) (Figure S13A). This trend was also observed in pose 96 from -294.65 kJ/mol to -201.1 kJ/mol (Figure S13B) and AlphaFold 3 from -169.861 kJ/mol to -123.471 kJ/mol (Figure S13E) predicted conformations, suggesting that alkaline conditions attenuating key electrostatic interactions are a general phenomenon, revealing that the local microenvironment may finely regulate the binding process. In pose 949(Figure S13C) and pose1124 (Figure S13D), the interactions between Arg730 and Arg731 and the protein TRPC4AP were relatively strong at pH 8.0.

In summary, these in-depth computational biology findings collectively elucidated that the key interactions at the TRPC4-TRPC4AP complex interface, which underpin H_2_-induced [Ca^2+^_i_]_t_, are not only structurally reasonable but also dynamically stable, energetically advantageous, and potentially subject to precise regulation by the physiological environment.

### Effects of H_2_ on intracellular cations

The TRP channel is a non-selective cation channel composed of four subunits, permeable to a variety of cations, including Ca^2+^, K^+^, and Na⁺ (Figure S4). These cations exhibit competitive interactions within the channel, with Ca^2+^ typically having higher permeability than Na⁺ and K^+^ 55.

To confirm the effects of H_2_ on sodium and potassium ions, we used Na⁺/K⁺ ion probes to investigate ion influx in MSCs (Figure S4). We found that upon adding H_2_ to the conventional culture medium, the cytoplasmic Na⁺ level increased slightly (Figure S4B). In contrast, K⁺ influx remained unchanged (Figure S4A). When the medium was switched to a Ca^2+^-free solution, Na⁺ influx continued to increase, whereas K⁺ influx showed no significant changes. These results suggested that H_2_ induces cation influx through TRPC4, with a much higher Ca^2+^ permeability than that of Na⁺ and K⁺. The slight increase in Na⁺ may be related to the NAX system, which helps expel excess Ca^2+^ that enters the cell. Similar phenomena were observed in HUVECs (Figures S2C-E).

### H_2_ triggers proton efflux, drives intracellular alkalinization, and elicits a Ca^2+^ signaling response in cells

**Figure 5:**
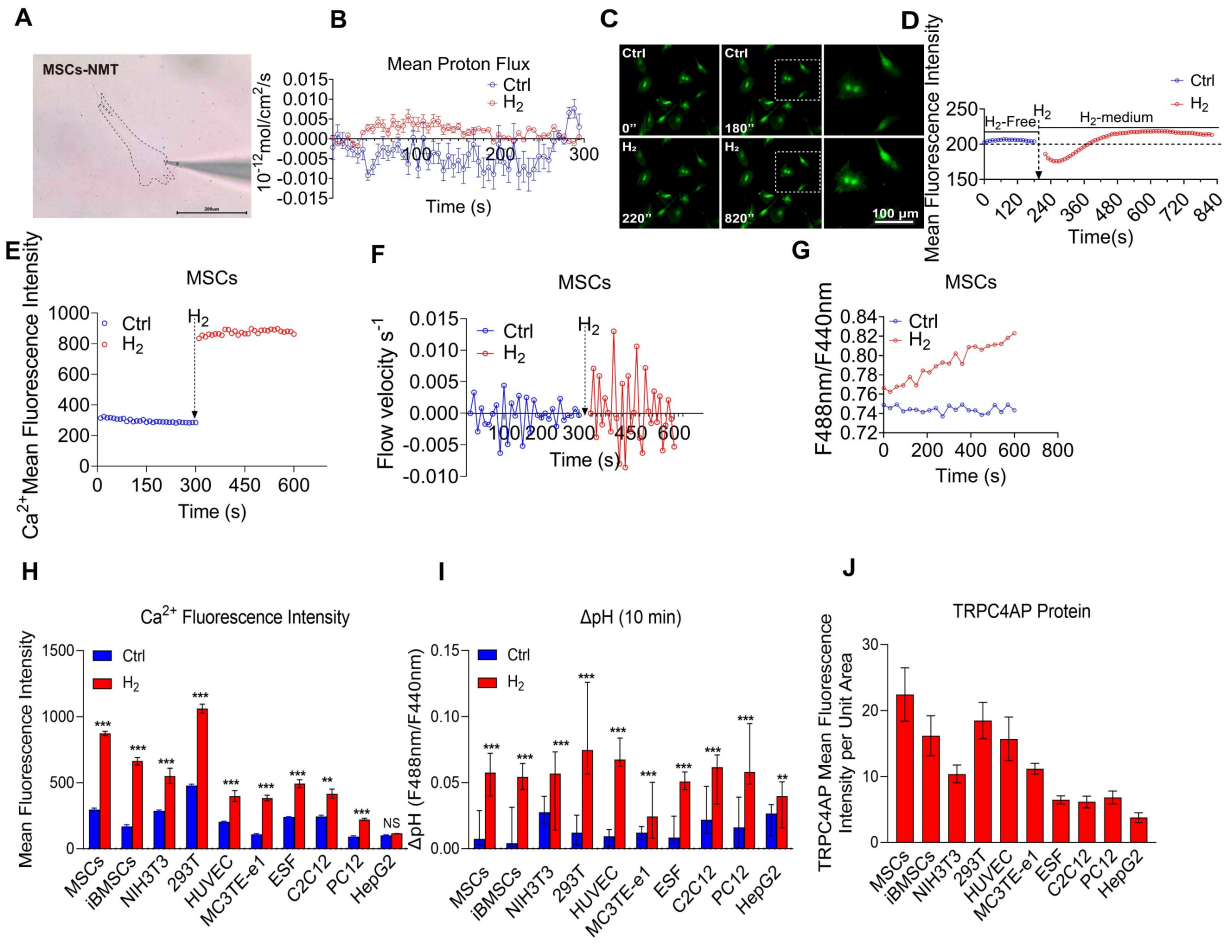
** H_2_ intervention on proton extrusion, intracellular pH dynamics, calcium transients in mesenchymal stem cells and multi-cellular calcium signaling pH dynamics, and TRPC4AP expression. A.** Schematic diagram of live cell NMT detection of MSCs. **B.** Dynamic changes in proton flux across the plasma membrane of MSCs under the influence of H_2_. **C** & **D.** Time-lapse imaging showing the effect of H_2_ on intracellular pH changes and mean fluorescence intensity statistics. **E**. Ca^2+^ relative fluorescence intensity statistics over 600 s in MSCs under H_2_ treatment. **F.** Ca^2+^ flow velocity measurements over 600 s in MSCs under the influence of H_2_. **G.** Intracellular pH variation in MSCs under the influence of H_2_.** H.** Ca^2+^ relative fluorescence intensity statistics in cells. **I.** Intracellular pH variation in cells. **J**. TRPC4AP mean fluorescence intensity per unit area in cells. Data in H, I and J were processed using an unpaired t-test, and were plotted as Mean ± SEM. *P value < 0.05; **P value < 0.01; ***P value < 0.001; no stars for P value > 0.05. Scale bar in A= 200 μm; Scale bar in C= 100 μm.

The H_2_-induced TRPC4-TRPC4AP binding site mainly comprises basic amino acids in the TRPC4 domain. In the extended exploration of this study, NMT analysis showed that H_2_ exposure led to proton efflux (Figure 5A), while PH fluorescence probes indicated an increase in intracellular pH (Figure 5C-D), creating an alkaline microenvironment. These conditions favor the stability of basic amino acids. The observations suggested that H_2_ may indirectly regulate TRPC4-TRPC4AP binding by altering the intracellular acid-base environment.

Initial observations in MSCs focused on dynamic changes in Ca^2+^ fluorescence intensity (Figure 5E), flow velocity (Figure 5F), and intracellular pH (Figure 5G) after H_2_ addition. To evaluate the broad applicability of this effect, parallel experiments and comprehensive analyses were conducted across multiple cell lines. The results indicated that hydrogen treatment not only induced [Ca^2+^_i_]_t_ (Figure 5H) but also consistently triggered an alkaline shift in the intracellular microenvironment across all tested cell types (Figure 5I). Furthermore, TRPC4AP expression levels varied among cell lines (Figure 5J, Figure S14), suggesting that its abundance may be linked to the specific responsiveness of cells to H_2_.

### *In vivo* validation of H_2_-induced intracellular Ca^2+^ elevation

**Figure 6:**
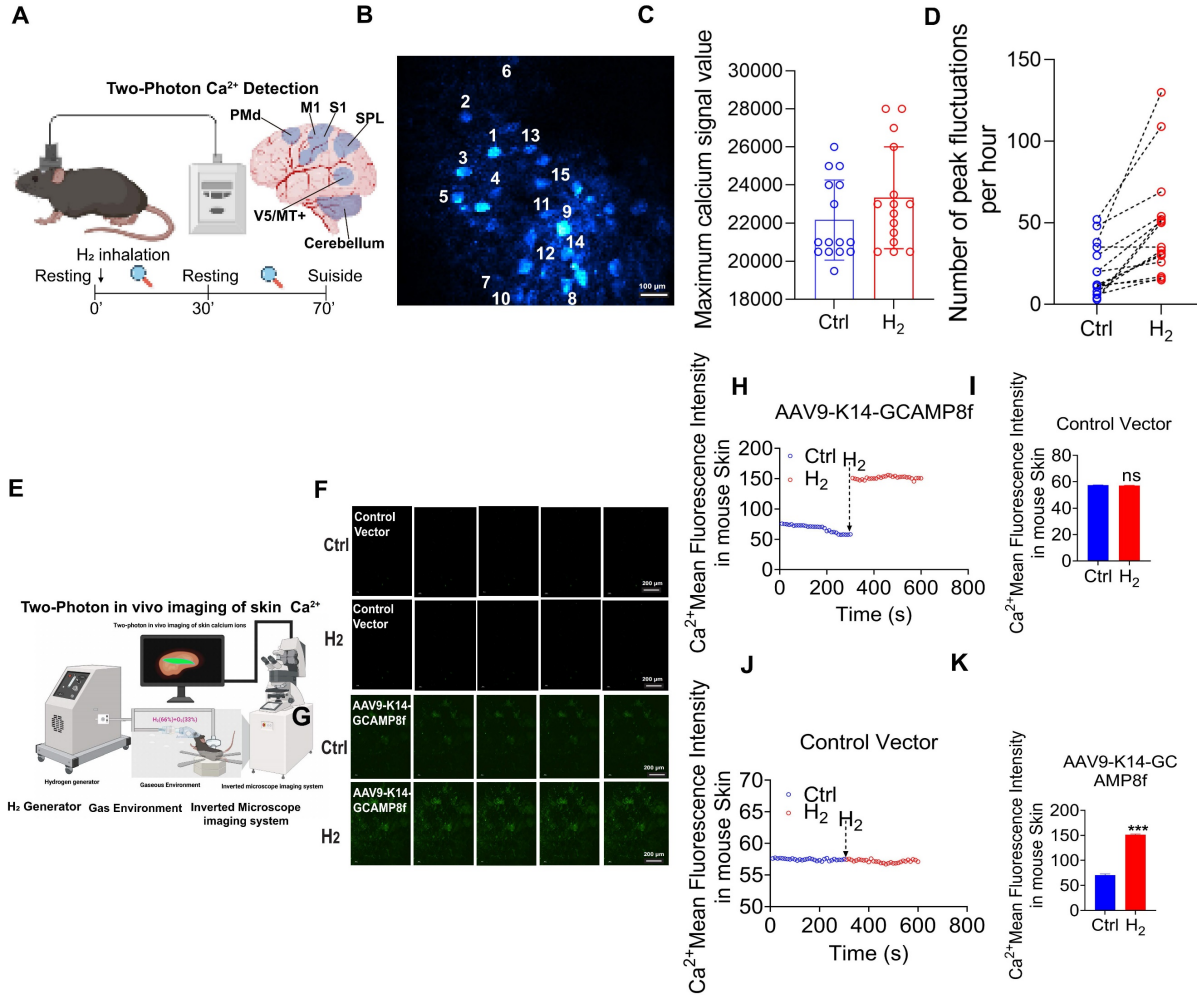
***In vivo* validation of H_2_-induced [Ca^2+^_i_]. A.** Upper left, common window of mouse brain preparations for *in vivo* two-photon Ca^2+^ imaging; upper right, six distinct functional areas of the mouse cerebral cortex, including the monitored Primary Motor Cortex (M1) region.; Schematic diagram of the animal experimental procedure. **B.** Randomly selected 15 neurons in the M1 region. **C.** Comparison between the control group (Ctrl) and H_2_ inhalation group (H_2_) in the number of peak fluctuations per hour. **D.** Comparison between Ctrl and H_2_ groups in the maximum calcium signal value. **E.** Two-photon *in vivo* imaging of skin calcium ions. **F&G.** Fluorescence images of Ca^2+^ at 0 and 600 s in empty vector (AAV9-K14-PS8) and GCAM plasmid-transfected group (AAV9- K14-GCAMP8f). **H&I.** Ca^2+^ mean fluorescence intensity in mouse skin over time and Ca^2+^ mean fluorescence intensity signal value in empty vector (AAV9-PS8-K14).** G&K.** Ca^2+^ mean fluorescence intensity in mouse skin over time and Ca^2+^ mean fluorescence intensity signal value in GCAM plasmid-transfected group (AAV9- K14-GCAMP8f). *P value < 0.05; **P value < 0.01; ***P value < 0.001; no stars for P value > 0.05. Scale bar in B= 200 μm; Scale bar in F and G= 200 μm.

To further validate the *in vivo* regulatory effect of H_2_ on intracellular Ca²⁺, two-photon *in vivo* calcium imaging was employed to monitor real-time calcium dynamics in the mouse brain and skin (Figure 6A). Adeno-associated viruses (AAVs) carrying the calcium indicators jGCaMP7 and GCaMP8f were stereotactically injected into the primary motor cortex (M1 region) and the dorsal skin of C57BL/6J mice to achieve neuron- and skin-specific expression. In the brain imaging experiments, quantitative analysis of 15 neurons within the M1 region revealed that H_2_ inhalation significantly increased the frequency of calcium transients compared to the control group, with averages of 50 and 25 transients per hour, respectively (Figure 6B-C). Furthermore, H_2_ exposure enhanced the maximum amplitude of calcium signals (Ctrl: 22,000 ± 500 vs. H_2_: 23,000 ± 450; Figure 6D). For skin imaging, a H_2_-enriched environment was established via nasal inhalation and dorsal H_2_ perfusion (Figure 6E). (time-lapse fluorescence imaging of skin *in vivo* (Figure 6F-G). In mice without the calcium indicator, no difference in dorsal calcium fluorescence was observed (Ctrl: 57.46± 0.15 vs. H_2_: 57.17 ± 0.23; Figure 6-I). In contrast, in the experimental group expressing the indicator, H_2_ inhalation induced a significant increase in calcium fluorescence within the specific labeled areas (Ctrl: 67.89± 6.45 vs. H_2_: 151.26 ±2.38; Figure 6G, Figure 6K). These *in vivo* findings were highly consistent with our *in vitro* observations, demonstrating that H_2_ effectively promoted [Ca^2+^_i_] elevation in both physiological and cellular systems.

### H_2_ enhances cell motility by inducing Ca^2+^ influx into the cells to restructure the cytoskeleton

**Figure 7:**
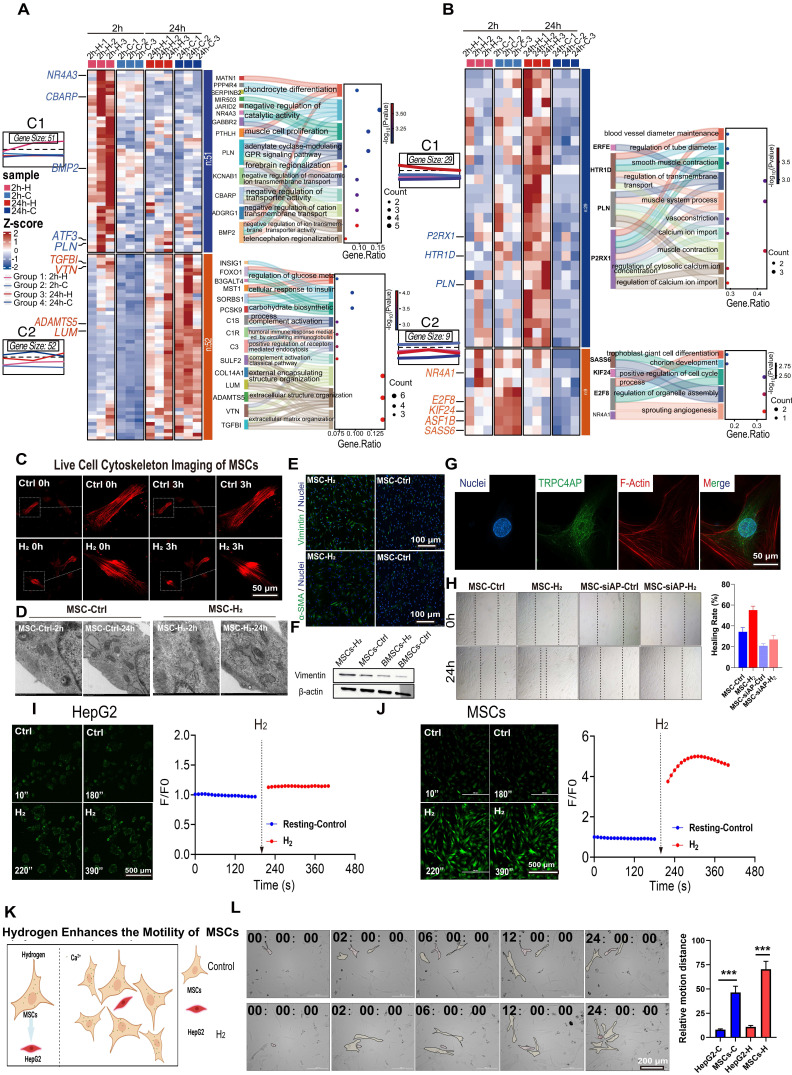
** H_2_ enhances cell motility by increasing [Ca^2+^_i_], thereby remodeling the cytoskeleton. A** & **B.** RNA sequencing analysis of cells treated with H_2_ for 2 h and 24 h. **C.** Time-lapse F-Actin probe images of MSCs in H_2_-free (Control) and H_2_-medium. **D.** Electron microscopy results of MSCs after treatment with H_2_ for 2 h and 24 h. **E.** Immunofluorescence results of Vimentin and α-SMA in the Control and H_2_ groups of MSCs. **F.** Western Blot results of the effect of H_2_ on Vimentin expression in MSCs and BMSCs. **G.** Fluorescence co-localization results of F-Actin (red) and the TRPC4AP (green). **H.** Cell scratch results and healing rate (right) for MSCs treated with H_2_ for 24 h after siRNA AP treatment. **I** & **J.** Time-lapse images of the [Ca^2+^_i_] changes in H_2_-free and H_2_-medium (red) in HepG2 (H), MSCs(I) and Fluo 4 averaged F/F0 trace in imaging H_2_-free (blue) and H_2_-medium (red). **K.** Schematic diagram of H_2_ enhancing the motility of MSCs.** L.** Detection of co-culture and corresponding migration distance of tumor cells HepG2 (red) and MSCs (yellow) in H_2_-free and H_2_-medium using a live-cell imaging system and relative motion distance. Data in H and L were processed using an unpaired T test, and were plotted as Mean ± SEM. *P value < 0.05; **P value < 0.01; ***P value < 0.001; no stars for P value > 0.05. Scale bar in C and G= 50 μm; Scale bar in E= 100 μm; Scale bar in I and J= 500 μm; Scale bar in L= 200 μm.

Zhao *et al.* previously found that H_2_ can enhance stem cells motility 9. Our time-series transcriptomic study revealed that H_2_-treated MSCs are regulated by a program centered on Ca^2+^, which is temporally synchronized in time with the H_2_-mediated reorganization of the cytoskeleton 9. RNA sequencing analysis of MSCs treated with H_2_ for 2 h (2h-H vs 2h-C) and 24 h (24h-H vs 24h-C) identified differentially expressed genes (DEGs) and revealed significant GO-BP enrichment patterns (Figures S9-S10). K-means clustering analysis of upregulated genes showed distinct temporal expression profiles. Genes elevated in the 2h-H group (Figure 7A) were associated with negative regulation of cation/calcium transmembrane transport genes (e.g. CBARP, PLN, KCNAB1), indicating feedback control of H_2_-induced [Ca^2+^_i_]_t_, prevent persistent overload. Meanwhile, extracellular matrix (ECM) remodeling genes (e.g., TGFB1, LUM, COL14A1) showed early activation (Figure 7B), indicating the transcriptional initiation of structural reorganization. By 24 hours, DEGs were enriched in cell contraction and calcium- dependent pathways (e.g., HTR1D, P2RX1), as well as proliferation-related processes, suggesting that calcium-mediated cytoskeletal remodeling is a key driver of MSCs migration.

Transcriptomic analysis of HUVECs conducted 2 h post H_2_ treatment (2h-H_2_ vs 2h-Ctrl) revealed significant inter-group differences, with an increased number of DEGs (Figure S7C-E). GO and KEGG enrichment analyses indicated upregulation of processes related to development and ion regulation, along with cytoskeletal gene enrichment and notable activation of the calcium signaling pathway (Figure S7F). Suggesting that H_2_ may modulate cytoskeletal remodeling in HUVECs via [Ca^2+^_i_]_t_. By 24 h, the upregulated genes were primarily associated with cytokine signaling. In contrast, genes related to [Ca^2+^_i_] were downregulated (Figure S7F), indicating a shift towards Ca^2+^ efflux or cessation of influx, thereby initiating downstream signaling cascades. These findings are consistent with the electron microscopy and protein expression results at different time points following H_2_ treatment. Further demonstrating the spatiotemporal coordination of H_2_ in calcium homeostasis and cytoskeletal dynamics.

Live-cell imaging over 3 h showed that, compared with the untreated control group, the contraction dynamics of the F-actin cytoskeleton in H_2_-treated MSCs were significantly accelerated (Figure 7C). Ultrastructural analysis by transmission electron microscopy (TEM) revealed that, after acute (2 h) and chronic (24 h) H_2_ exposure, microfilament bundles in MSCs aggregated near the cell membrane (Figure 7D). Protein expression analysis showed that, 24 h after H_2_ treatment, the expression of mesenchymal markers in MSCs was upregulated, with increased levels of vimentin and α-smooth muscle actin (α-SMA) (Figure 7E-F).

Immunofluorescence results indicated that TRPC4AP in MSCs was primarily localized in the cytoplasm, with no colocalization observed with the F-actin cytoskeleton network (Figure 7G). Functional assessment using a scratch assay showed that H_2_ significantly enhanced MSCs migratory capacity, with a wound closure rate of 58% in the treated group after 24 h, compared to 38% in the control group (Figure 7H). The effect of H_2_ was validated in the HUVECs, demonstrating that H_2_ similarly enhances the migratory capabilities of cells, evidenced by accelerated wound healing speed (Figure S7A) and rapid reorganization of the cytoskeleton (Figure S7B). This pro-motility effect was significantly attenuated by TRPC4AP gene knockdown, which reduced [Ca^2+^_i_], confirming that H_2_-induced [Ca^2+^_i_]_t_ could enhance stem cells motility (Figure 7H). While histamine (a calcium agonist positive control) induced moderate [Ca^2+^_i_] (Figure S6-B) and upregulated vimentin (Figure S6C), α-SMA (Figure S6D), and collagen I (Figure S6E) expression, its efficacy remained inferior to H_2_ stimulation. Our results suggested that H_2_ regulates cellular motility function through TRPC4AP-mediated [Ca^2+^_i_].

We further verified whether cell motility is related to Ca^2+^, by using Ca^2+^ live-cell imaging to screen for two cell types, one sensitive and one insensitive to H_2_-induced [Ca^2+^_i_]. H_2_ exhibited specificity among different cell types. MSCs showed a significant [Ca^2+^_i_] peak value of F/F₀ = 4.488 ± 0.389, while HepG2 tumor cells displayed a peak F/F₀ value of 1.143 ± 0.005 (Figure 7I-7J). The immunofluorescence assay demonstrated that TRPC4AP expression was significantly lower in HepG2 cells than in MSCs (Figure S11). By coculturing MSCs and HepG2 cells and analyzing cell movement distances (Figure S15), we confirmed that H_2_ selectively enhances MSC motility without affecting HepG2 cells (Figures 7K-L). This suggested that the mechanism underlying H_2_-induced [Ca^2+^_i_] is cell-specific and depends on TRPC4AP expression.

## Discussion and Conclusion

Our study provided evidence that H_2_ acts as a novel gaseous signaling molecule, specifically regulating [Ca^2+^_i_]_t_ by targeting the TRPC4-TRPC4AP axis. Traditional calcium transients refer to rapid increases and subsequent decreases in intracellular Ca^2+^ concentration over a short period of time 28. This phenomenon is typically triggered by the opening of Ca^2+^ channels in the cell membrane, allowing Ca^2+^ to flow into the cell and causing a temporary rise in intracellular Ca^2+^ concentration 56. Subsequently, Ca^2+^ pumps, Ca^2+^ carriers, and other mechanisms transport Ca^2+^ out of the cell or resequester it into intracellular calcium stores (such as the ER or sarcoplasmic reticulum), restoring the Ca^2+^ concentration to baseline levels 57, 58. In cardiomyocytes and skeletal muscle cells, [Ca^2+^_i_]_t_ triggers myofilament sliding, leading to muscle contraction. In nerve cells, [Ca^2+^_i_]_t_ is involved in synaptic transmission and promotes the release of neurotransmitters. Moreover, as a second messenger, Ca^2+^ activates a variety of downstream signaling pathways, regulating gene expression, cell cycle, cell differentiation 59, and other processes 60.

In this study, the [Ca^2+^_i_]_t_ induced by H_2_ is significantly different from traditional [Ca^2+^_i_]_t_ and represents a novel H_2_-dependent type of [Ca^2+^_i_]_t_. Parameters such as the amplitude, frequency, and duration of [Ca^2+^_i_]_t_ can accurately reflect the intensity and characteristics of intracellular calcium signals. H_2_ can induce reversible elevations in [Ca^2+^_i_]_t_ in various types of cells, a process that exhibits marked cellular heterogeneity. Specifically, different cell types exhibit inconsistent intensities of intracellular calcium signals after H_2_ stimulation, and there are also significant differences in the duration of Ca^2+^ signal peaks among cells.

In MSCs, exposure to H_2_ can trigger a rapid increase in cytosolic Ca^2+^ concentration within 30 s, which is comparable to that induced by calcium ionophores, and this signal exhibits a strict H_2_ dose-dependent characteristic. It is worth noting that after the H₂ treatment is stopped, the signal spontaneously returns to baseline levels in about 50 min and reappears with periodic oscillations. Compared with the calcium overload toxicity caused by ionomycin, cell viability remains normal after 24 h of H_2_ treatment, indicating its biosafety. It is important to note that the [Ca^2+^_i_]_t_ induced by H_2_ is characterized by rapidity and dependence on extracellular calcium, with minimal contribution from intracellular calcium stores such as the ER and Mito. This mechanism is distinct from the mode of action of traditional [Ca^2+^_i_]_t_.

The TRPC4-TRPC4AP complex is the core molecular target for H_2_-regulated calcium signaling 61, 62. Transmembrane calcium channel screening shows that although T/L/N-type VGCC and STIM1/Orai pathways are highly expressed in MSCs, only TRPC4/5-specific antagonists can significantly inhibit H_2_-induced [Ca^2+^_i_]. Unlike traditional [Ca^2+^_i_]_t_, the contribution of calcium stores to Ca^2+^ accumulation is relatively small, and extracellular Ca^2+^ plays a key role in the H_2_-induced [Ca^2+^_i_] process 63, 64. The absence of TRPC4 or TRPC4AP eliminates the calcium regulatory effect of H_2_, whereas reconstituting wild-type TRPC4 or TRPC4AP restores this function, establishing the necessity of the TRPC4-TRPC4AP axis for calcium signaling. To date, only the TRPC4-TRPC4AP complex has been shown to trigger calcium load when the ER-Ca^2+^ store is depleted, indicating that this pathway may regulate [Ca^2+^_i_] 62. As the most abundant regulatory protein in the TRP family, the functional loss of TRPC4AP leads to complete blockage of calcium signaling, suggesting that it may dominate channel activity through structural specificity. H_2_ may directly act on the signaling axis. However, other calcium channels that H_2_ may rely on also need to be confirmed in future studies, such as the subtle contribution of ER-Ca²⁺ stores and other potentially underlying calcium channels.

The dual arginine motif (Arg730/Arg731) in the CIRB domain at the carboxyl terminus of TRPC4 is the key binding site for TRPC4AP. The double-site mutation (R730A/R731A) leads to a complete loss of H_2_-induced [Ca^2+^_i_]. The CIRB functional box is found only in the TRPC4 subtype, and acts as a hub for H_2_ signaling determining the channel's specific response to H_2_.

This region is adjacent to or overlaps with the previously reported "rib-helix" domain, which mediates the Ca^2+^-CaM-dependent feedback inhibition 65. This spatial proximity strongly suggests that H_2_ may directly act upon or allosterically modulate this critical interface, thereby interacting with the classical CaM regulatory pathway or, co-opting this regulatory node in a novel manner to gate the channel open. Furthermore, TRPC4's interactions with other proteins, such as NHERF 66, 67, and its lipid-mediated regulatory mechanisms, provide a potential complex regulatory context for the broad and differential responses to H_2_ that we observed across various cell types.

At the structural biology level, the recently resolved cryo-EM structures of TRPC4 in its closed state provided an indispensable template for precisely mapping the H_2_ interaction site 68, 69. Our molecular docking and subsequent mutational validation efforts, built directly upon these structural foundations, allowed us to translate the abstract concept of " H_2_ sensing" into specific atomic interactions. Our study definitively identified the allosteric pocket within the S1-S4 voltage-sensing-like domain and the adjacent CIRB domain as the structural basis for H_2_-mediated regulation. Future research should systematically elucidate the molecular mechanisms underlying H_2_-regulated calcium signaling. These include resolving the open-conformation structure of TRPC4 to reveal the H_2_-induced allosteric gating mechanism; defining the assembly patterns and interface characteristics of the heterotetrameric TRPC1/4 complex that governs calcium dynamics; and employing multidisciplinary techniques to map the TRPC4-TRPC4AP interaction interface and quantify its remodeling by H_2_. These investigations will establish a comprehensive structure-function framework to explain how H_2_ encodes calcium-based cellular information.

We found H_2_ can induce proton extrusion (Figure 5A, B), which might result in an alkaline intracellular microenvironment in many cell types (Figure S12). We propose that H_2_ may affect the conformation of the TRPC4-TRPC4AP complex by regulating intracellular acid-base balance, thereby influencing the strength of TRPC4-TRPC4AP binding (Figure S13) and Ca^2+^ influx (Figure 5 H, I). The attenuation of interaction forces under pH 8.0 conditions may be associated with the maintenance of calcium homeostasis.

Zhang *et al.* have reported that H_2_ promotes proton extrusion to accelerate the growth of the lower hypocotyl of the mung bean 4. This pH-dependent regulatory mechanism may be related to the yet-to-be-discovered hydrogenase activity in eukaryotes. Given that H_2_ molecules can directly enhance the activity of various enzymes, we speculate that H_2_ may directly participate in the interaction between TRPC4-TRPC4AP, and these findings warrant further investigation. We propose that TRPC4AP may function as a regulatory switch for H_2_-induced [Ca^2+^_i_], and its activity could be modulated by the intracellular acid-base equilibrium.

Another key observation in this study is the heterogeneous responsiveness of different cell types to molecular hydrogen. We hypothesize that divergent TRPC4AP expression levels across cell lines and tissues (Figures 5H-J; Figure S8) generate distinct feedback strengths on hydrogen-evoked Ca^2+^ influx. Furthermore, cell-type-specific signaling landscapes, including basal reactive oxygen species levels, antioxidant capacity, and proton-pump activity—may cooperatively set the sensitivity threshold to H_2_ exposure. Elucidating this cell-type-specific response mechanism is a critical direction for future research. Comparative proteomic profiling integrated with functional readouts across multiple cell lines should provide a systematic approach to address this question.

In this study, we propose that the novel [Ca^2+^_i_]_t_ induced by H_2_ occurs as follows. During normal periodic calcium oscillations of the cells, the introduction of H_2_ opens the TRPC4-TRPC4AP channels on the cell membrane, allowing Ca^2+^ to rapidly influx from the extracellular space into the cytoplasm in a H_2_-dependent manner. As the local H_2_ concentration decreases, the intracellular Ca^2+^ concentration declines gradually, and the cells resume traditional calcium oscillations to maintain calcium homeostasis.

Our research has shown that H_2_-induced [Ca^2+^_i_]_t_ can enhance cellular motility and induce transcriptional reprogramming, and that these effects rely on TRPC4AP. H_2_ can modulate cellular biological functions by regulating intracellular Ca^2+^, which offers a novel research direction for the discovery of H_2_-based medicines. By integrating human gut metagenomic data (average daily H_2_ production of 0.2-1.5 liters) 70, a physiological correlation map has been established between endogenous H_2_ levels and calcium homeostasis. These explorations will lay the foundation for the development of new regenerative therapies based on gas signal programming.

Multiple clinical studies have confirmed the favorable safety profile of H_2_ when administered by inhalation, orally, or by injection 2, 71, 72. The discovery of its direct regulation of calcium signaling suggests that in cardiovascular and cerebrovascular diseases characterized by disrupted calcium homeostasis, H_2_-induced calcium influx may affect vascular tone or electrophysiological stability. This possibility warrants further validation in relevant disease models. Future preclinical studies and clinical trial designs should fully account for factors such as formulation, concentration, timing, and disease-specific pathology, while strengthening the monitoring of related functional indicators. This approach will help systematically define its therapeutic window and advance H_2_ therapy toward more precise and safe applications.

Our study has elucidated the molecular mechanism by which H_2_ functions as a novel gaseous transmitter that regulates ion channels, demonstrating its capacity to induce novel [Ca^2+^_i_]_t_ through the TRPC4-TRPC4AP axis, suggesting a mechanism of action distinct from those of other gasotransmitters. For instance, H_2_S is widely reported to activate channels such as TRPA1 via direct sulfhydration 73, 74, CO commonly regulates TRPC6 indirectly through the cGMP-PKG pathway 75, 76. Nitric oxide (NO) can also influence calcium channels via cGMP-dependent pathways or protein nitrosylation 76, 77. Our data demonstrated that the effect of H_2_ does not rely on these classical pathways but instead points to a novel mechanism mediated by the arginine-rich motif 730Arg-731Arg within the CIRB domain of TRPC4, a region critical for TRPC4-TRPC4AP association. This interaction could evoke conformational rearrangements that enhance extracellular Ca^2+^ influx, while preserving global Ca^2+^ homeostasis by generating a weakly alkaline cytosolic environment, ultimately remodeling the cytoskeleton. This is consistent with the recently proposed concept of "ion channel-effector signalosomes," exemplified by the "redox-calcium cycle" microdomain formed by TRPV4 and NOX2 78. Thus, H_2_ functions as a novel endogenous gaseous signaling molecule with gasotransmitter activity, regulating calcium signaling networks via the TRPC4-TRPC4AP pathway. Future studies should systematically screen for and validate additional molecular targets of H_2_ to construct a more comprehensive H_2_ signaling network.

## Methods

### Ca^2+^, K^+^, Na^+^ ion probe staining and imaging in live cells

Cells were seeded on glass coverslips in a culture dish to reach the desired confluence. The cells were washed gently with warm Hank's Balanced Salt Solution (HBSS) (Thermo Fisher Scientific 14175095) to remove the culture medium and incubated with ion-sensitive fluorescent probes for Ca^2+^-Fluo-4-AM (5 μM ) diluted in serum-free medium (SFM) (KeyGEN, KGAF024), K^+^-EPG-4-AM (5 μM) diluted in SFM (Maokang MX4521), for Na^+^-ENG-2-AM (5 μM ) diluted in SFM, and for Ca^2+^-Rhod-2-AM (5 μM ) diluted in HBSS (YEASEN 40776ES72) for 10-30 min at 37°C to allow probe uptake. After incubation, the excess probes were washed off with HBSS or SFM, and the cells were placed in a recording chamber for microscopy. A confocal microscope equipped with a CCD camera (C2, Nikon) was used to capture the fluorescence signal, which indicated ion concentration. The cells were maintained at 37°C and the atmosphere (CO_2_ level) was controlled throughout the imaging process to keep the cells viable and monitor calcium dynamics in response to stimuli. Fluorescence intensity changes were analyzed to assess ion activity within the cells.

### Animal studies

Male C57BL/6J mice (8 weeks old) were obtained from Charles River Laboratories (Beijing). Mice were maintained in a 12-hour light/dark cycle at 25 °C with free access to food and water. All animal experiments were approved by the Institutional Animal Care and Use Committee (IACUC) and were conducted in accordance with the guidelines for the ethical treatment of animals (Approval No. KWT-240529). To monitor Ca^2+^ influx in the primary motor cortex (M1), mice were stereotactically injected with adeno-associated virus (AAV) expressing Syn1-driven jGCaMP7 (AAV-Syn1-jGCaMP7). To monitor Ca^2+^ influx in the dermal layer of the back skin, mice were intradermally injected in the back with a 3×2 cm matrix pattern using an adeno-associated virus expressing GCaMP8f driven by the K14 promoter (AAV9-K14-GCaMP8f) 79.

Mice were anesthetized with 2% isoflurane in oxygen and positioned in a stereotactic frame. The scalp was shaved and disinfected with ethanol. For mice used in brain imaging, a midline incision was made, and a small craniotomy (1-2 mm in diameter) was performed at the coordinates (X=2 mm lateral, Y=0.8 mm anterior, Z=2.4 mm depth) relative to bregma, to target the primary motor cortex (M1). AAV-Syn1-jGCaMP7 (titer: 1 × 10^13^ vg/mL) was injected at a rate of 0.2 μL/min using a microinjection pump (UltraMicroPump3 (UMP3)). A total of 50 μL of AAV vector was delivered into M1. For mice used in dorsal skin imaging, AAV9-K14-GCaMP8f virus (titer: 1 × 10^7^ vg/mL) was injected at a rate of 20 microliters per square centimeter using a microliter syringe, delivering a total of 120 microliters of the viral vector into each 2×3 cm matrix. The needle was left in place for an additional 5 min to allow for viral diffusion, and the incision was closed with sutures. Mice were allowed to recover for 2-3 weeks to ensure proper viral expression and cellular uptake.

### Two-photon microscopy for *in vivo* Ca^2+^ imaging in the primary motor cortex (M1) and dermal layer of the back skin

#### Experimental Setup and Preparation

To monitor real-time calcium dynamics in the primary motor cortex dermal layer of the back skin of C57BL/6J mice infected with AAV-Syn1-jGCaMP7 and AAV9- K14-GCAMP8f, *in vivo* two-photon imaging was performed as follows:

#### Animal Preparation

After the recovery period post-surgery and sufficient expression of jGCaMP7 in the M1 region and GCaMP8f in the dermal layer of the back skin, mice were anesthetized with isoflurane (2% for induction, 1-1.5% for maintenance) and placed onto a custom imaging platform. Core body temperature was maintained at 37 °C using a heating pad.

#### Cranial Window and Imaging Setup

A cranial window above the primary motor cortex was prepared by carefully removing the overlying skin and gently clearing the bone over M1. A round coverslip was placed on the exposed cortical surface to protect the brain tissue and reduce motion artifacts. The window was sealed with dental cement (C&B Metabond) to secure the coverslip.

#### Imaging Preparation

For brain imaging, the mice were placed under the objective lens of the two-photon microscope (Olympus FV1000, Leica SP8), and the brain region of interest was positioned in the imaging field. The system was equipped with a Ti:sapphire laser for two-photon excitation (900 nm), and the emitted fluorescence from jGCaMP7 was collected through a 510-550 nm bandpass filter. For the dorsal skin imaging, the mouse was placed under the objective lens of the two-photon microscope (Nikon AX), and a hydrogen-enriched environment was established through nasal inhalation and dorsal hydrogen perfusion. The fluorescence emitted by GCaMP8f was collected via a 510-550 nm channel.

### Lipofectamine 2000 transfection of siRNA

Cells were seeded at a density of 5×10^3^ cells/well in a 96-well plate and cultured in standard growth medium for 24 h. On the day of transfection, cells were seeded in growth medium without antibiotics to achieve 30-50% confluence. SiRNA was diluted in serum-free Opti-MEM (Gibco, 2898884), mixed gently, and Lipofectamine 2000 (Thermo Fisher Scientific, 11668019) was diluted in Opti-MEM. The siRNA and Lipofectamine 2000 were mixed and incubated at room temperature for 20 min to form siRNA-Lipofectamine 2000 complexes. The siRNA-Lipofectamine 2000 mixture was added to each well, and the plate was gently rocked back and forth to mix. Cells were incubated in a 37 °C CO_2_ incubator for 48-96 hours, and the medium was changed after 6-8 h.

### Cell viability assay

Cell viability was assessed using the Cell Counting Kit-8 (CCK-8). The cells were seeded at a density of 5×10^3^ cells per well in a 96-well plate, and grown for 24 h in standard growth medium, which was then replaced with medium saturated with hydrogen gas. Cell viability was assessed using the CCK-8 reagent at 2, 4, 8, and 24 h. The optical density (OD) was measured at 450 nm using a Perkin Elmer microplate reader.

The blank group contained only culture medium, the control group contained cells not treated with saturated H_2_, and the positive control group contained cells treated with 2µM ionomycin to induce calcium overload.

Cell viability was determined as Abs of the experimental group-Abs of the blank group)/Abs of the control group-Abs of the blank group ×100%.

### Cell death rate assessment and live cell imaging

NucGreen Dead 488 Ready Probes Reagent (ThermoFisher Scientific S7020) is a membrane-impermeable stain that selectively labels cells with compromised membranes by binding to DNA and emitting a vivid green fluorescence, without penetrating or affecting the integrity of living cells. This reagent is used to quantify the relative rate of cell death, as per to the guidelines provided by Thermo Fisher Scientific.

Images of 293T cells were captured using the Cytation 5 Cell Imaging Multimode Reader (Biotek Instruments, Inc., Winooski, VT, USA). Cells were treated with NucGreen Dead 488 Ready Probes Reagent to determine the numbers of live and dead cells after hydrogen treatment at 2, 4, 8, and 24 h. The cell imaging data were processed and cell counts were analyzed using Gen5™ Data Analysis Software (Bad Friedrichshalle, Germany).

The control group contained cells that were not treated with saturated hydrogen gas, and the positive control group contained cells treated with 75% ethanol.

### Cell scratch assay

Cells were seeded in a 6-well plate, and once confluent, the medium was removed and the cell layer was gently scratched uniformly across the well with a pipette tip to create a 'wound.' The cells were washed with PBS to remove any debris and floating cells. Fresh culture medium was added, and the plate was placed in the Live Cell Imaging System (Biotek Instruments, Inc., Winooski, VT, USA). Images of the wound area were automatically captured at the start and at regular time intervals. After the experiment, the images were analyzed to measure the rate of cell migration into the wound area over time. The wound-healing rate was quantified by comparing wound areas at different time points, reflecting cell migration and proliferation.

### Western blotting

Cells were washed, harvested, and centrifuged, and proteins were extracted using a Cell Protein Extraction Kit (San Gong, C006225). All procedures were carried out on ice. After cell lysis, protein quantification was performed using the BCA Protein Assay Kit (Beyotime P0010). Equal amounts of cell protein samples were separated by 10% sodium dodecyl sulfate-polyacrylamide gel electrophoresis (SDS-PAGE) and transferred onto a nitrocellulose membrane (Millipore). The membrane was blocked with a 3% BSA for 2 h at room temperature, then washed three times with TBST, and incubated with the target primary antibody overnight at 4 °C. After incubation with a fluorophore-conjugated secondary antibody for 1 h in the dark, the bands were visualized and captured using the LI-COR® Odyssey Infrared Imaging System.

### Molecular docking and molecular dynamics

The initial structure of TRPC4 was obtained from the PDB database under entry 7B0J. In the absence of experimentally resolved TRPC4AP structures, we employed AlphaFold to predict the full-length of this associated protein. we used SWISSMODEL to refine and complete the TRPC4 protein chain, thereby ensuring its functional integrity. Subsequently research, the binding modes and detailed interactions between TRPC4 and TRPC4AP were investigated using protein-protein docking technology.

We generated 2,000 potential docking configurations between TRPC4 and TRPC4AP. The PSC (Pairwise Shape Complementarity) scoring system software 80, 81, was used to perform an automated clustering and scoring of these configurations, identifying the top 60 TRPC4 and TRPC4 AP binding modes 81, 82. These initial docking models were based on a rigid docking approach. In an effort to refine these docking configurations, we employed energy optimization to eliminate potential geometric conflicts, laying a robust foundation for further experimental validation and clinical applications.

In addition, by utilizing advanced protein-docking software, we simulated the binding process between TRPC4 and TRPC4AP and examined the specific interactions at their interfaces.

In a practical application of AlphaFold 3, we submitted the amino acid sequences of TRPC4 and TRPC4AP into the AlphaFold 3 platform to obtain a model of the TRPC4-TRPC4AP complex. We analyzed the interfacial interactions within this complex. Notably, an interaction between arginine at position 730 of TRPC4 and glutamic acid of TRPC4AP, which received the highest score using the PSC. We have obtained results consistent with our previous protein-protein docking, confirming the accuracy of our analysis.

The docked structure was solvated using GROMACS to obtain a protein complex in an aqueous environment. Following energy minimization, a short molecular dynamics simulation was conducted (simulation temperature: 303.15 K, force field: CHARMM36). The simulation trajectory was extracted to calculate the binding free energy between the proteins using gmx_MMPBSA 54.

The protonation states of specific amino acids in the protein were analyzed and adjusted (protonated or deprotonated) using propka3 software in conjunction with the CHARMM-GUI online tool 83, 84. Short molecular dynamics simulations were then performed under at pH 7.4 and pH 8.0 using GROMACS 85. The simulation trajectories were extracted to calculate the interaction forces between arginine residues at positions 730 and 731 on TRPC4 and the TRPC4AP.

### Construction and identification of pX459-sgRNA knockout plasmids targeting TRPC4 and TRPC4AP

Based on genomic information for the TRPC4 and TRPC4AP genes from NCBI, the exons were annotated. The nucleotide sequences of the first and second exons of the target genes were entered into the sgRNA online design website http://crispr.mit.edu. Two sgRNA sequences with the lowest off-target rates were selected, synthesized by Suzhou Hongxun Biotechnology Co., Ltd., and named TRPC4 sgRNA-1/TRPC4 sgRNA-2 and TRPC4 AP sgRNA-1/TRPC4 AP sgRNA-2. The sgRNA primers were annealed to form double-stranded DNA through a programmable annealing process. The vector pX459 was linearized after digestion with BbsI enzyme. Subsequently, T4 DNA ligase was used to ligate the annealed sgRNA double-stranded DNA to the linearized pX459 at room temperature to obtain recombinant plasmids, which were then sequenced and verified for correctness before use in subsequent experiments.

### Preparation and selection of 293T cells with TRPC4 and TRPC4AP gene knockout

HEK293T cells were seeded into a 6-well plate. At 80% confluency, the cells were transfected with the pX459 plasmid carrying TRPC4 and TRPC4AP sgRNA. The empty plasmid pX459 was used as a negative control. After 36 h, pressure screening was performed with 1 μg/mL puromycin. Following two rounds of puromycin selection, once the control group cells had all died, the experimental group cells were digested into single cells. Subsequently, the limited dilution method was used to isolate single cells and expand the culture. The cells were passaged, frozen, and stored for future use.

### Identification of gene and protein knockout in 293T cell lines with TRPC4 and TRPC4AP gene knockout

Monoclonal cell strains were collected and genomic DNA was isolated using a DNA extraction kit. Based on the genomic sequences of TRPC4 and TRPC4AP targeted by sgRNA, specific primers were designed based on the intron sequences flanking the corresponding exons and used for PCR amplification (Novoprotein, E035). The amplified products were sequenced and aligned to detect the knockout effect of the target genes in the monoclonal cell strains.

### Bulk-RNA sequencing and analysis

MSCs were subjected to H_2_-enriched or control medium for 2 h and 24 h, yielding four independent groups (2 h- H_2_, 2 h-Ctrl, 24 h- H_2_, 24 h-Ctrl; n = 3 per group). Total RNA was extracted and submitted for paired-end RNA-seq (Novogene, Beijing). Libraries were constructed with an insert size of ~300 bp and sequenced on an Illumina NovaSeq 6000 platform, yielding ≥ 60 million reads per sample (≈6 Gb raw data). Clean reads were obtained by removing adapters, low-quality bases (Phred < 20) and rRNA contaminants using fastp (v0.23). Alignment to the human reference genome (GRCh38) was performed with HISAT2 (v2.2.1), followed by gene-level quantification using StringTie (v2.1) with Ensembl annotation. Normalized expression values (FPKM) were calculated in R (v4.4.1) with the Ballgown package. Differential expression was evaluated by DESeq2 (v1.38) with Benjamini-Hochberg FDR < 0.05 and |log₂FC| ≥ 1. All downstream analyses, including PCA, clustering and visualization, were conducted in R (v4.4.1). The script utilized in this study is provided in the Supplemental Materials. Differentially expressed genes were screened based on the criteria of an adjusted P-value < 0.05 and an absolute fold change > 2. Principal component analysis (PCA) was conducted to assess overall variance in the dataset, and hierarchical clustering was performed to group samples based on their gene expression patterns. Heatmaps of the differentially expressed genes were generated using the pheatmap package. Gene Ontology (GO) and Kyoto Encyclopedia of Genes and Genomes (KEGG) enrichment analyses were performed on differentially expressed genes using the clusterProfiler package, to identify biological processes and pathways enriched in the data.

To facilitate the clustering and visualization of time-series gene expression data from RNA-Seq experiments, we introduced the ClusterGVis package. This tool provided a streamlined and efficient solution for analyzing time-series gene expression data in a single, easy-to-execute step 86.

In detail, we conducted individual K-Means fuzzy clustering analyses to delineate the transcriptomic profiles of DEGs in MSCs exposed to H_2_ for 2 h (MSCs-2 h- H_2_) versus control conditions (MSCs-2 h-Ctrl), and after 24 h (MSCs-24 h- H_2_) versus control conditions (MSCs-24h-Ctrl). Subsequently, we performed an integrated K-Means clustering on the top 5000 DEGs identified from the aforementioned pairwise comparisons to elucidate the overarching transcriptional dynamics. GO enrichment analysis was subsequently applied to each distinct cluster to identify significantly enriched functional annotations and biological processes. Furthermore, we conducted a temporal transcriptome analysis to track the evolution of gene expression patterns over the specified time intervals, providing insights into the temporal regulation of MSCs in response to H_2_. Data processing in HUVEC RNA-seq followed the same route.

### Detection of intracellular pH under H_2_ exposure

BCECF-AM is a fluorescent dye capable of crossing cell membranes. BCECF-AM itself is non-fluorescent but, upon entering cells, is hydrolyzed by intracellular esterases to form BCECF, which is retained within the cells. The fluorescence intensity of BCECF is sensitive to intracellular pH, making it suitable for detecting cellular pH changes.

A total of 5,000 cells were seeded in a black-walled, clear-bottom 96-well plate and incubated overnight for 24 h to allow cell attachment. Cells were loaded with BCECF-AM (2.5 µM) in serum-free medium at 37 °C for 30 min, the H_2_-treated group was incubated in saturated hydrogen-containing medium. After loading, cells were washed several times with fresh, dye-free medium and further incubated at 37 °C for 15-30 min to ensure complete hydrolysis of the AM ester by intracellular esterases, generating responsive BCECF.

Fluorescence intensity was measured using a microplate reader at dual excitation wavelengths of 488 nm and 440 nm, with emission detected at 535 nm. Changes in intracellular pH were reflected in the F488nm/F440nm ratio.

### Cellular immunofluorescence assay

Cells were cultured on glass coverslips in a 6-well plate to the desired confluence. The medium was aspirated, cells were washed with PBS, and fixed with 4% paraformaldehyde for 15 min at room temperature. Subsequently, cells were permeabilized with 0.1% Triton X-100 for 10 min, then blocked with 3% BSA in PBS for 1 h, and incubated with the primary antibody overnight at 4 °C, followed by three washes with PBS. The fluorophore-conjugated secondary antibody was then added for 1 h at room temperature in the dark, followed by another wash with PBS. The nuclei were stained with DAPI for 5 min and coverslips were mounted on slides with the mounting medium. The slides were protected from light to prevent photobleaching and the cells were examined by confocal fluorescence microscopy (Nicon, C2, Japan).

### Preparation of cell samples for TEM

The cultured cells were harvested and centrifuged to form a pellet, then immediately fixed in a solution of 2% glutaraldehyde and 2% paraformaldehyde in PBS for 2 h at 4 °C. The pellets were washed with sodium cacodylate buffer, then post-fixed in 1% osmium tetroxide. The samples were dehydrated through an ascending ethanol series, transitioned with propylene oxide, embedded in resin, and polymerized at 60-70 °C. The embedded blocks were trimmed and sectioned with an ultramicrotome to obtain ultrathin sections, which were then stained with uranyl acetate and lead citrate, mounted on copper grids, and examined using a transmission electron microscope (HITACHI, HT7700, Japan).

### Analysis of calcium transient signals and extraction of kinetic parameters

The analysis of calcium transient signals and extraction of kinetic parameters were performed according to the following steps: (a) Signal Acquisition: Raw fluorescence signals were obtained using Nikon confocal analysis software. (b) Signal Normalization: To eliminate background interference, all raw signals were converted to change ratios relative to the baseline (F/F₀). (c) Baseline Definition: The baseline fluorescence (F₀) was specifically defined as the average fluorescence intensity during the pre-stimulation time window in live cells, once a stable state had been reached. (d) Dynamic Signal: Real-time fluorescence intensity (F) was recorded at a frequency of H_2_ for subsequent analysis.

### Statistical analysis

Statistical analyses were performed using GraphPad Prism 9.0. Comparisons between two groups were analyzed using a two-tailed Student's t-test. Comparisons among multiple groups were analyzed using one-way analysis of variance (ANOVA) followed by Tukey's post-hoc test. All P-values were subjected to appropriate correction for multiple comparisons where applicable.

## Supplementary Material

Supplementary figures and tables.

## Data Availability

All other data are available in the article and its Supplemental files or from the corresponding author upon reasonable request.
