# Supplementary material for: Molecular hydrogen triggers TRPC4-TRPC4AP-dependent reversible calcium transients via extracellular influx
Source: Theranostics. 2026 Feb 26;16(9):4843–64. doi: 10.7150/thno.124352 (PMC12964245; doi:10.7150/thno.124352)
Supplement: Supplementary file 1 — Supplementary figures and tables. [file thnov16p4843s1.pdf]

Supplemental Materials

Molecular hydrogen triggers TRPC4-TRPC4AP-dependent reversible calcium transients via extracellular influx

Pengxiang Zhao[1,*], Han Li[1, *], Zisong Cai[1, *], Xujuan Zhang[1], Xiaohu Wen[1], Ziyi Liu[1], Shihao Jiang[1], Xue Jiang[1], Jiateng Wang[1], Zheng Dang[1], Mengyu Liu[1], Fei Xie[1], and **Xuemei Ma[1, #]**

[1]College of Chemistry and Life Science, Beijing University of Technology, Beijing 100124, P. R. China

# Correspondence: Xuemei Ma, xmma@bjut.edu.cn

*** Pengxiang Zhao, Han Li ,and Zisong Cai, contributed equally as first authors**

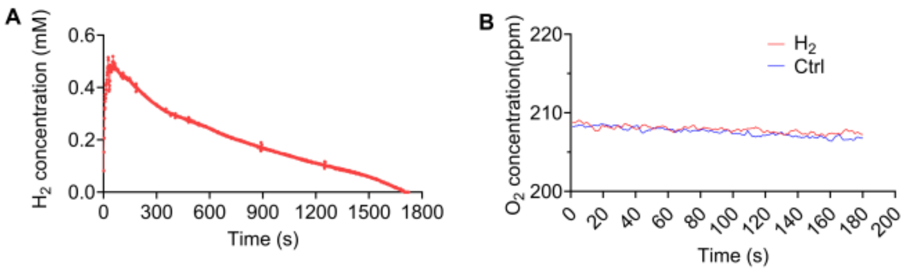

Figure S1. Real-time monitoring of H_2 and O_2 levels in the culture medium after a single administration of H_2.

(A) Real-time monitoring curve of H_2 concentration.

(B) Real-time monitoring curve of O_2 concentration.

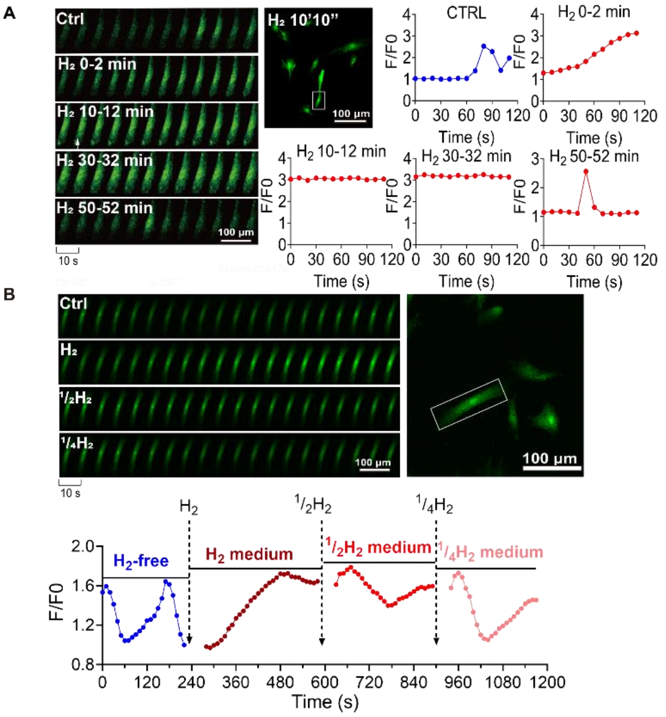

Figure S2. H_2 Induces reversible and concentration-dependent calcium oscillations in MSCs.

(A) Different time periods of Fluo-4 images and averaged F/F0 traces in H_2-induced MSCs [$Ca^{2+}{}_i$] during extended time-lapse imaging.

(B) Time-lapse Ca^{2+} images and Fluo-4- averaged F/F0 trace of MSCs in H_2-free, H_2-medium (Saturated H_2 medium), 1/2H_2-medium, and 1/4H_2 medium. Scale bar in A and B= 200 μm.

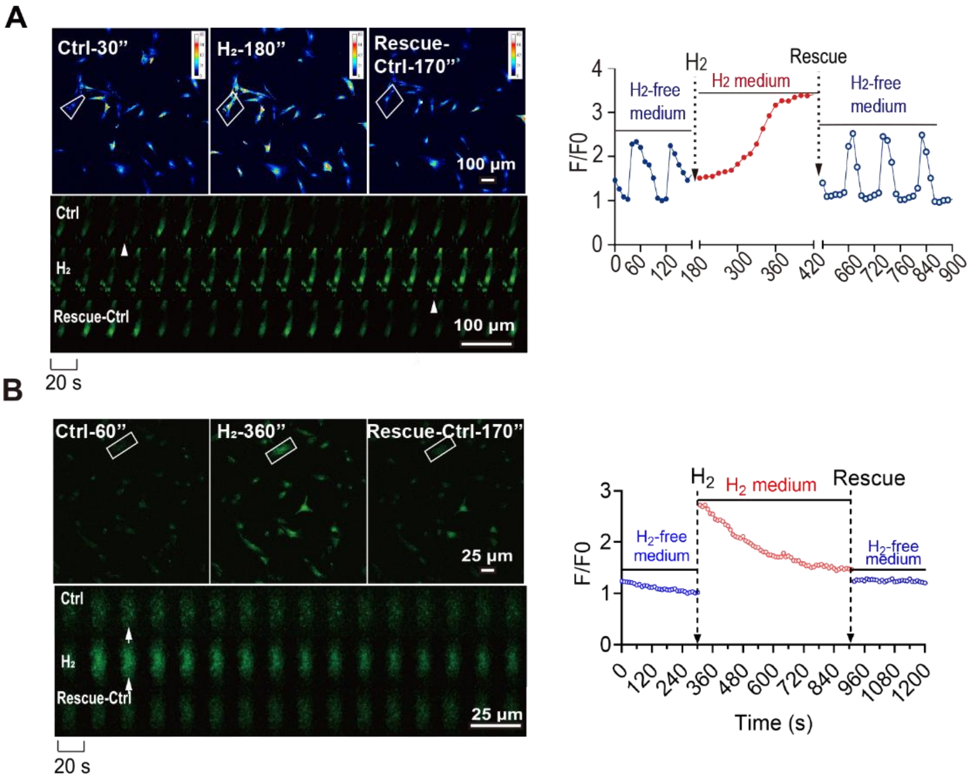

Figure S3. H_2 Induces Intracellular Calcium Elevation in MSCs and HUVECs.

(A) Pseudocolor (A left, upper) and time series (A left, lower) images and the Fluo-4 averaged F/F0 trace of the [$Ca^{2+}{}_i$] changes in H_2-free, H_2-medium, and then back to H_2-free conditions.

(B) Time-lapse (B) images and the Fluo 4 averaged F/F0 trace of the [$Ca^{2+}{}_i$] changes in H_2-free, H_2-medium, and then back to H_2-free conditions in HUVEC. Scale bar in A = 100 μm. Scale bar in B = 25 μm.

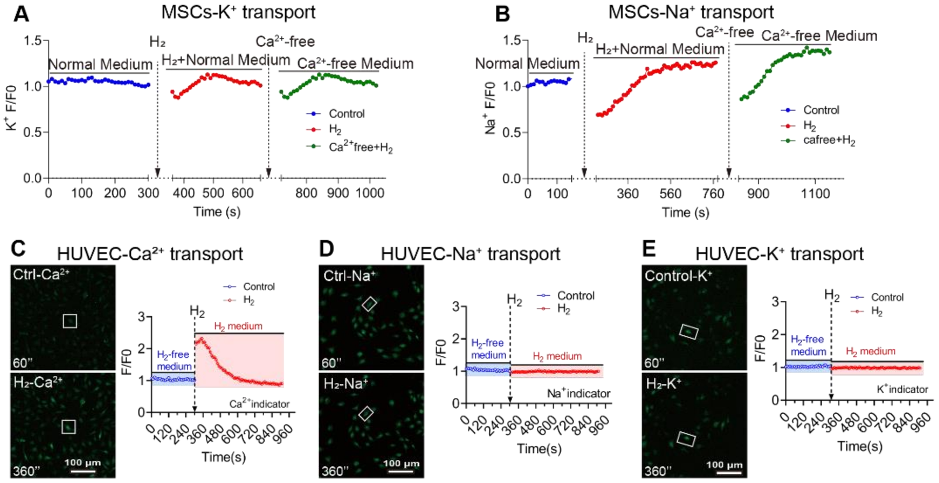

Figure S4. Time-lapse-averaged F/F0 traces showing the influence of H_2 on the influx of different cations in MSCs and HUVECs.

(A) & (B) H_2-induced K^+ and Na^+ transport under normal-medium, H_2-medium and Ca^{2+}-free medium conditions in MSCs.

(C)-(D) & (E) Comparison of the effects of H_2 on Ca^{2+}, K^+, and Na^+ influx in HUVEC cells. Scale bar in C,D and E= 100 μm;

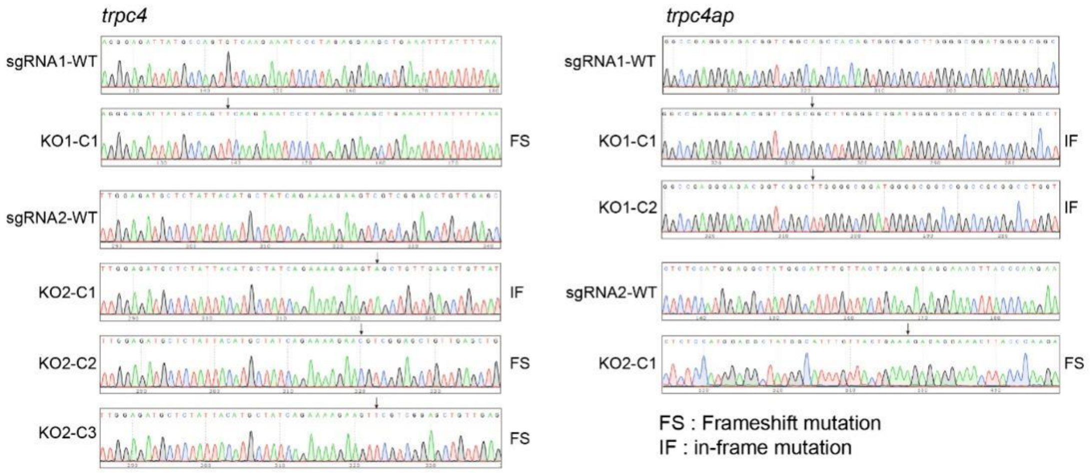

Figure S5. Validation of TRPC4 and TRPC4ap gene knockdown in monoclonal 293T cell lines.

The mutant site is indicated by an arrow. Wild-type (WT) sequences of the corresponding exons were determined by Sanger sequencing and are presented with chromatograms. For each detected sequence variant, the detection frequency and the type of mutation are shown.

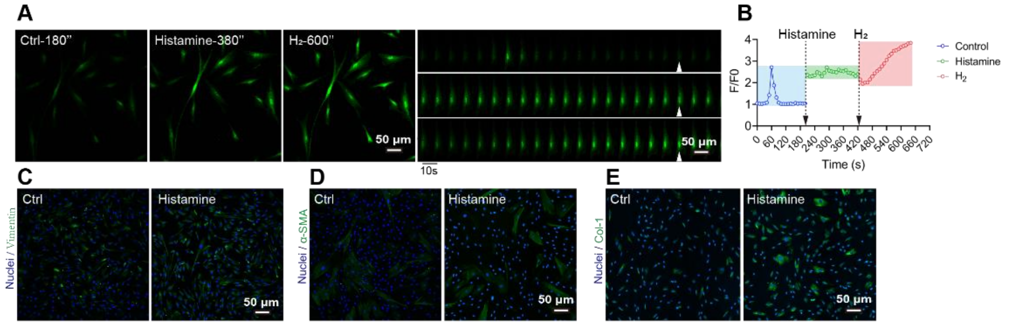

Figure S6. Comparative analysis of the effects of histamine, a Ca^{2+} agonist, and H_2 on [$Ca^{2+}{}_i$] in MSCs, and their impact of histamine on the cytoskeleton.

(A) & (B) Time-lapse (A) images and the Fluo4 averaged ΔF/F0 trace (B) of the [$Ca^{2+}{}_i$] changes under H_2-free, Histamine, and then H_2 conditions in MSCs.

(B) - (E) Immunofluorescence results of Vimentin, α-SMA, and Collagen-1 in the Control and Histamine groups of MSCs. Scale bar = 50 μm;

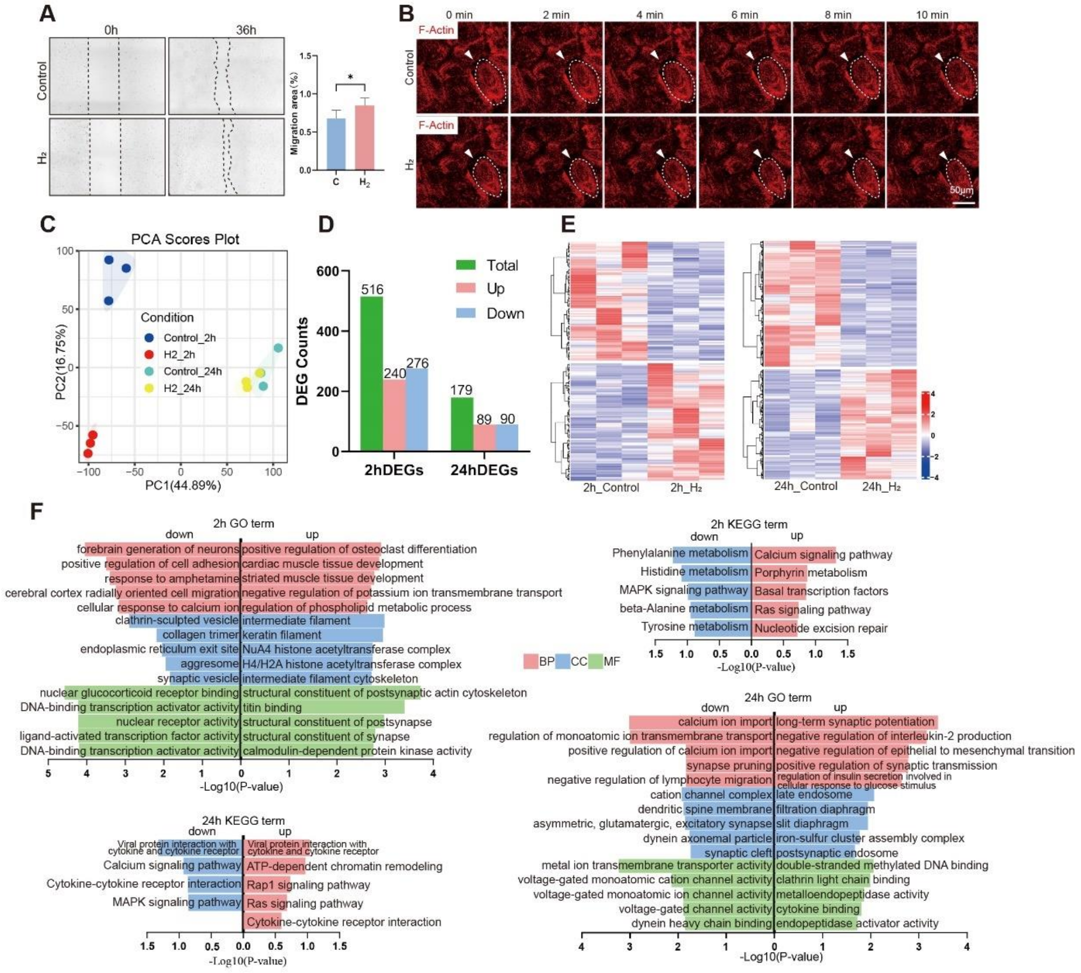

Figure S7. Impact of H_2 on the migratory function of HUVEC cells and its effects on

transcription.

(A) Effect of H_2 on the motion ability of HUVECs.

(B) Impact of H_2 on the cytoskeletal structure of HUVECs.

(C) PCA diagram of sequencing data of each group.

(D) Differentially expressed genes (DEGs) count results.

(E) Hierarchical clustering heat map of DEGs in HUVECs following 2 h and 24 h H_2 treatment.

(F) H_2-2h vs Control-2h and H_2-24h vs Control-24h column charts for GO enrichment analysis and a histogram of KEGG enrichment analysis. For B, using normal culture medium as the control group, changes in the cytoskeleton were captured over a 10-minute period. The medium was then immediately replaced with saturated hydrogen culture medium, to ensure consistent visibility, and changes were recorded for an additional 10 minutes. In the control group, there were no significant changes in the cytoskeleton during the initial 10 minutes. However, after switching to saturated hydrogen culture medium for 10 minutes, intercellular spacing increased, and the cytoskeleton changed, showing a contraction trend. Data A were processed using an unpaired t-test, and were plotted as Mean ± SEM. *P value < 0.05. Scale bar in B= 50 μm;

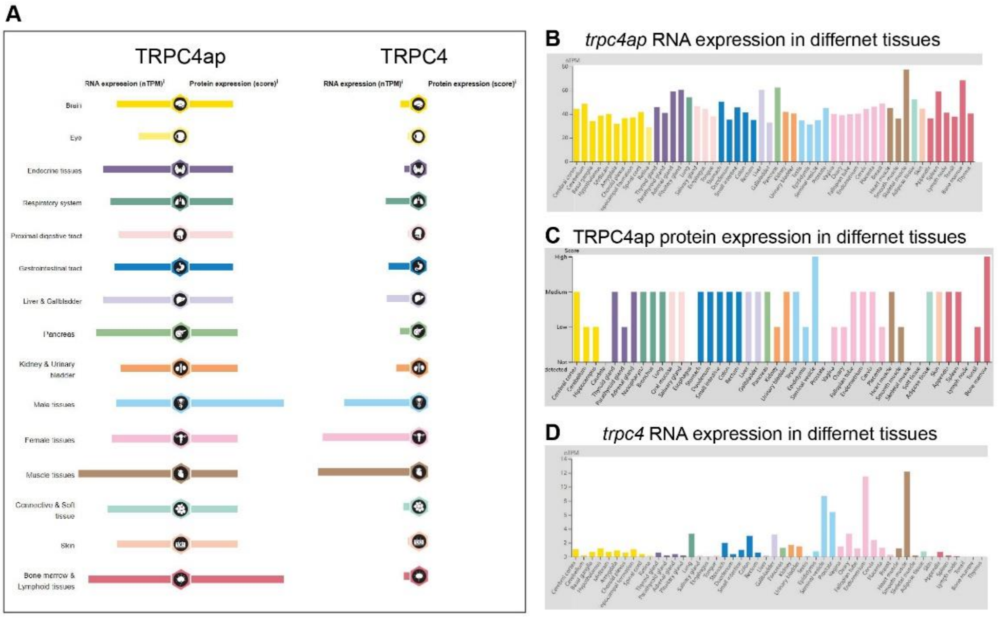

Figure S8. Overview of TRPC4 and TRPC4ap expression based on data from the Human Protein Atlas.

(A) Expression levels of TRPC4ap and TRPC4 across various human organs, with RNA levels shown on the left and protein levels on the right.

(B) and (D) RNA expression patterns of TRPC4ap and TRPC4 in distinct tissue types. (C) Distribution of TRPC4ap protein expression across multiple tissues. The images have been sourced, downloaded, and reorganized from the following URLs:

https://v19.proteinatlas.org/ENSG00000100991-TRPC4AP/tissue

https://v19.proteinatlas.org/ENSG00000133107-TRPC4/tissue

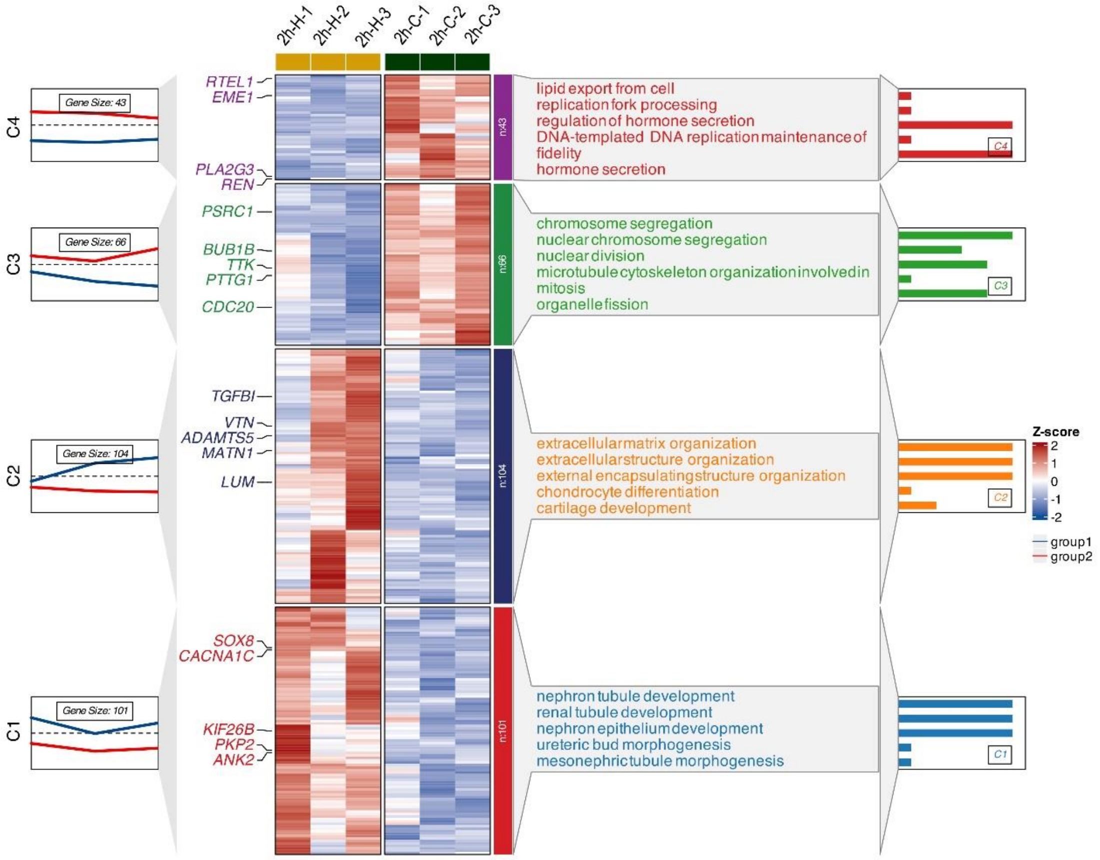

Figure S9. Expression trends, heat maps, and significantly enriched Biological Process terms for each cluster of DEGs in MSCs after H_2 treatment for 2 h.

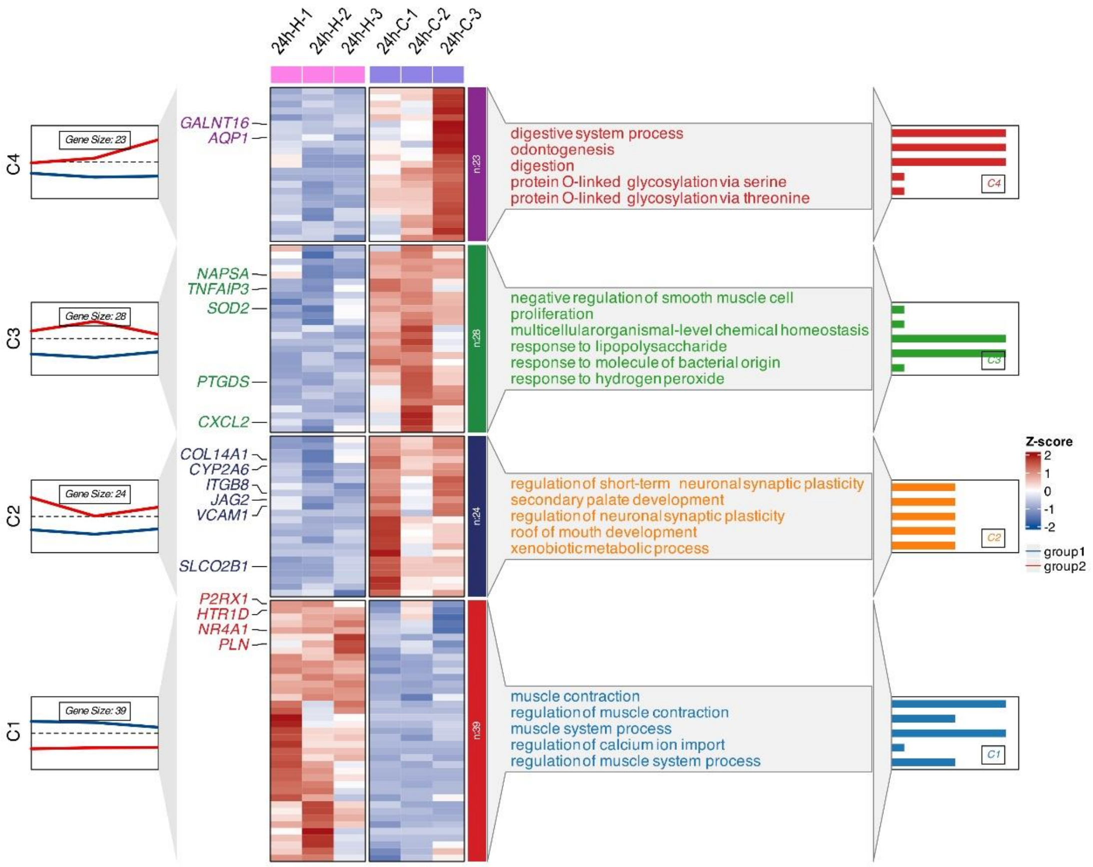

Figure S10. Expression trends, heat maps, and significantly enriched Biological Process terms for each cluster of DEGs in MSCs after H_2 treatment for 24 h.

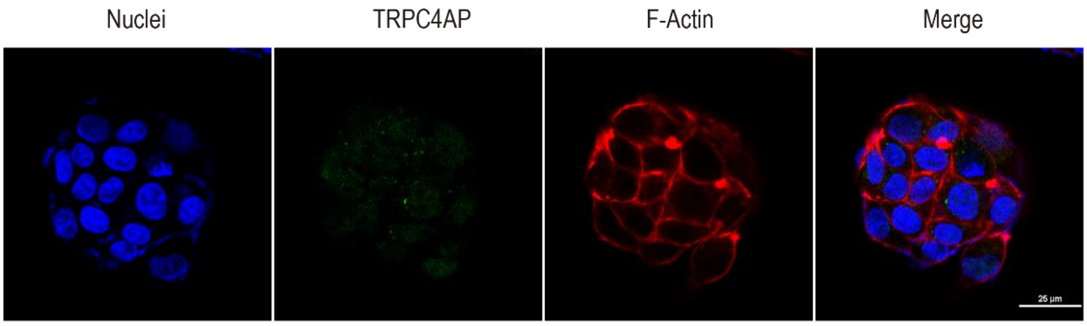

Figure S11. Fluorescence co-localization results of F-Actin (red) and the TRPC4AP (green) of HepG2. Scale bar = 25 μm;

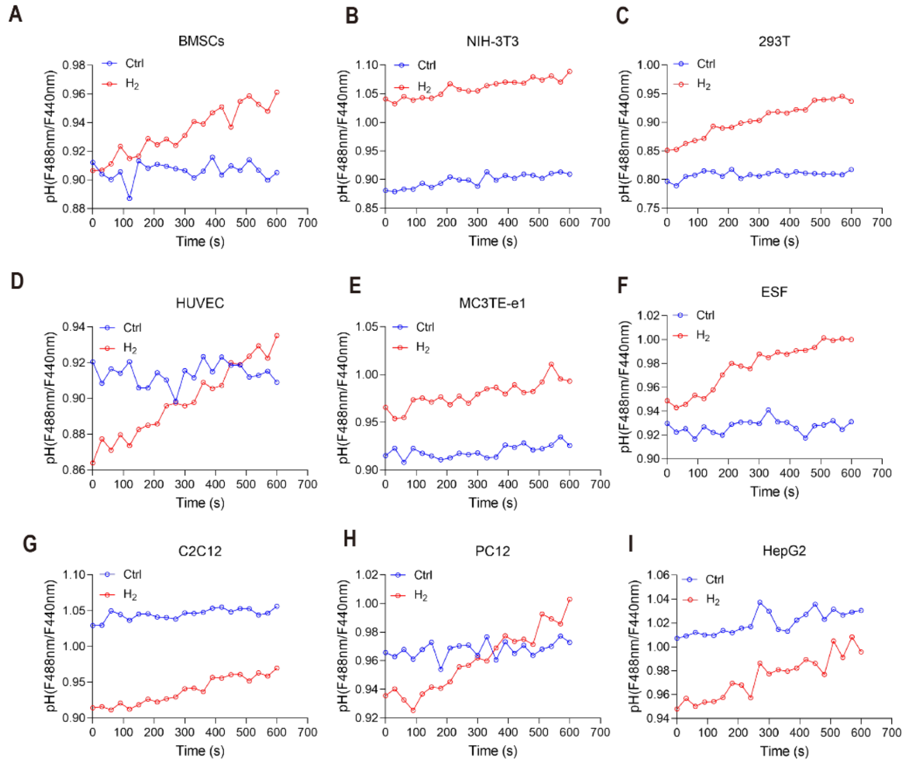

Figure S12 . Intracellular pH variation under the influence of H_2 in different cells.

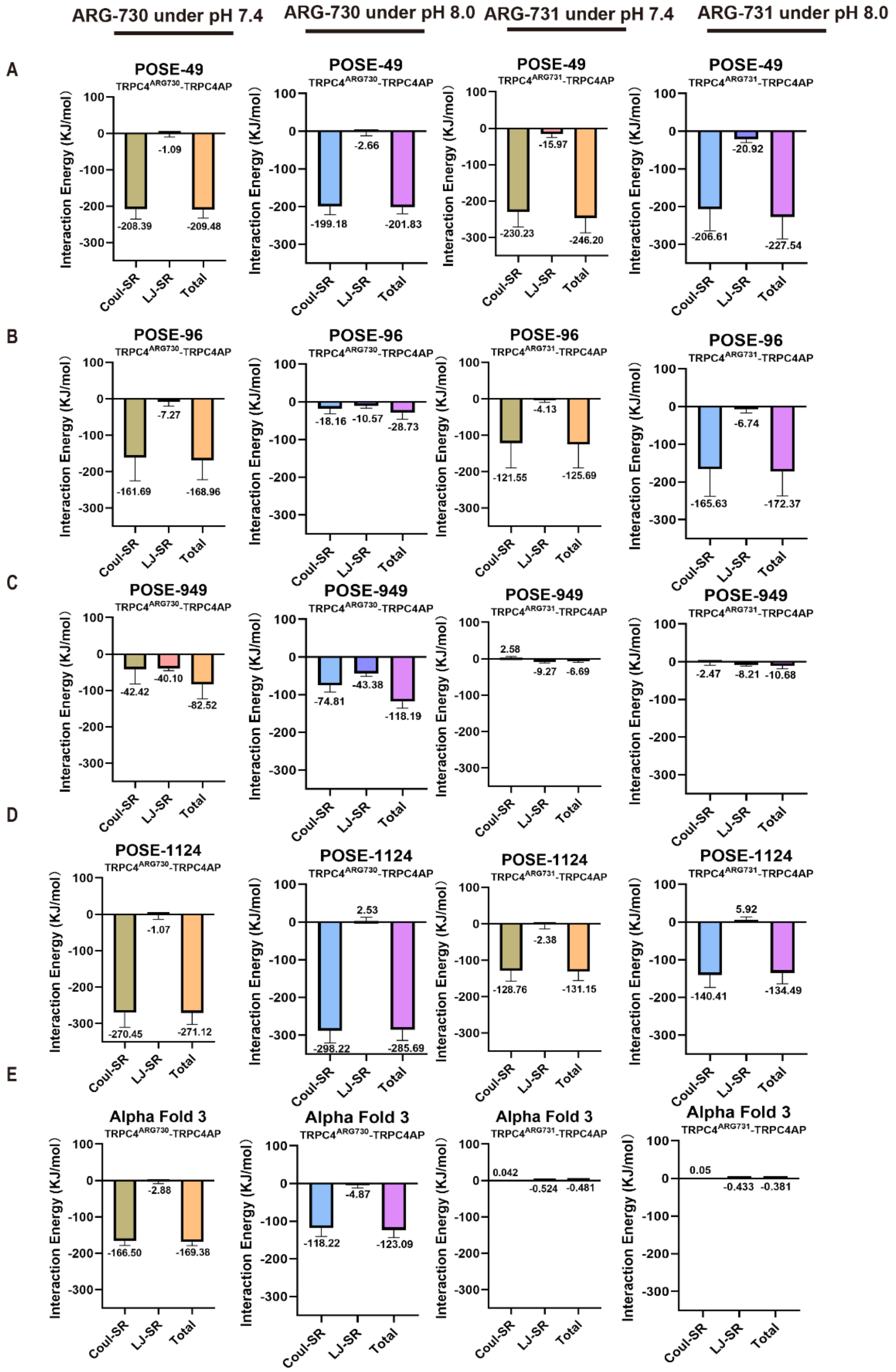

Figure S13. Interaction forces between arginine residues at positions 730 and 731 of the TRPC4 protein and the TRPC4AP protein in protonated states under pH 7.4 and pH 8.0 in molecular dynamics simulations.

(A).pose-49;(B).pose-96; (C).pose949; (D).pose1124.(E) .Alpha Fold 3. From left to right: Interaction forces between TRPC4, specifically the arginine at position 730, and the TRPC4AP protein at pH 7.4; Interaction between TRPC4, specifically the arginine at

position 730, and the TRPC4AP protein at pH 8.0; Interaction between TRPC4, specifically the arginine at position 731, and the TRPC4AP protein at pH 7.4; Interaction between TRPC4, specifically the arginine at position 731, and the TRPC4AP protein at pH 8.0. Coul-SR (short-range Coulomb force, representing electrostatic interactions), LJ-SR (short-range Lennard-Jones potential, representing van der Waals forces), and Total (total interaction force).

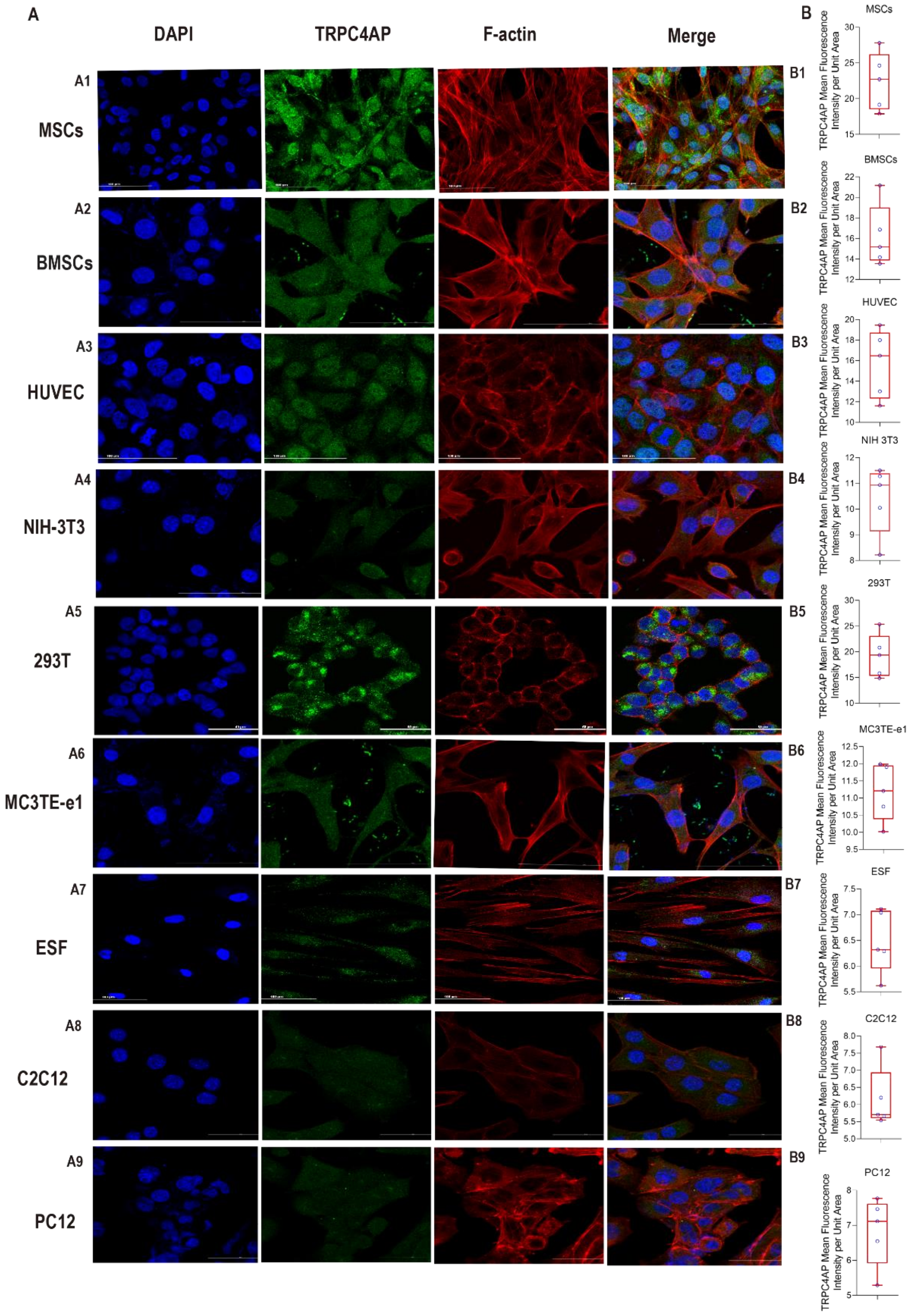

Figure. S14. Fluorescence co-localization results of F-Actin (red) and TRPC4AP (green) in cells. Scale bar = 50 μm;

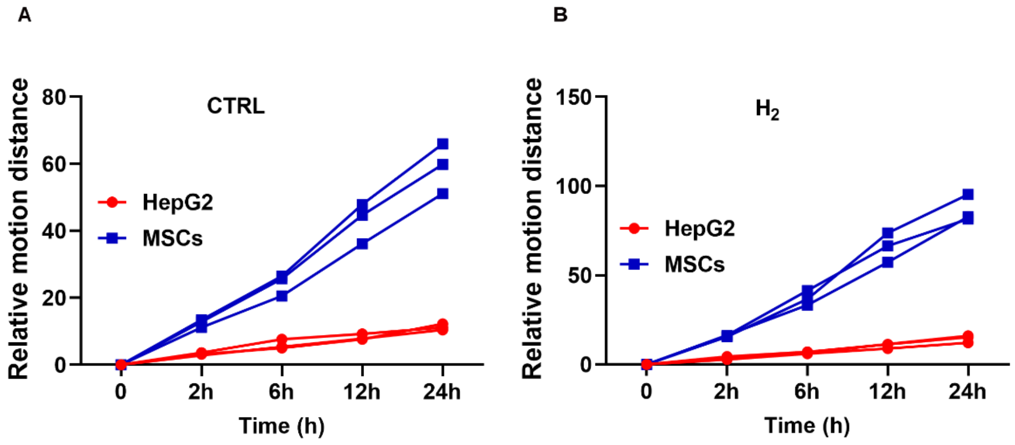

Figure S15. Corresponding migration distance of tumor cells HepG2 and MSCs under (A).H_2-free and (B).H_2-medium using a live-cell imaging system .

TABLE

Table S1. Comparative analysis of Ca^{2+} response to H_2 stimulation across different cell types.

Cell Types	F_0	Peak F/F_0	F(average) under H_2	Flow velocity amplitude	Flow velocity amplitude under H_2
MSCs	296.07	2.819	873.59	0.0107	0.0216
BMSc	167.80	3.908	662.63	0.0373	0.0870
HUVEC	202.59	1.700	399.51	0.0135	0.0330
293T	478.88	2.035	1061.49	0.0112	0.015
NIH3T3	286.72	1.67	552.34	0.0075	0.0209
MC3TE-e1	108.04	3.675	384.39	0.0296	0.0631
ESF	240.63	1.908	492.54	0.0045	0.0245
C2C12	242.92	1.725	418.82	0.0141	0.0162
PC12	90.04	2.512	222.46	0.0460	0.0587

Table S2 Materials and Ragents

RAGENT or RESOURSE	SOURCE	IDENTIFIER
**Antibodies**		
Rabbit anti-TRPC4AP	Proteintech	29310-1-AP
Mouse anti-αSMA	Cell SignalingTechnology	19245
Rabbit anti-Vimentin	Cell SignalingTechnology	5741
Mouse anti-Beta actin	Proteintech	66009-1-1g
Rabbit anti- Collagen Ⅰ	Abcam	AB254113
secondary antibodies (Alexa Fluor 488)	Cell SignalingTechnology	4412
**Chemicals**		
Ca^{2+}-GPCR,Fluo-4,AM (5μM,for Live-cell)	KeyGEN	KGAF024
Na^{+}-ENG-2,AM, (5 μM, for Live-cell)	Maokang	MX4514
K^{+}-EPG-4,AM, (5 μM , for Live-cell)	Maokang	MX4521
DAPI	ThermoFisher	R37606
ER-tracker (1 μM)	YEASEN	40764ES20
Mito-tracte r(100 nM)	YEASEN	40741ES50
Stem cell culture-medium	iCell	PriMed-icell-012-sf
JC-1 (2 μM)	ThermoFisher Scientific	T3168
CCK-8	Beyotime	C0038
Trypsin EDTA(0.25%)	ThermoFisher Scientific	25200056
DMEM high	Gibco	11865092
1640 culture-medium	Gibco	11875119
Ca^{2+}-free medium	iCell	PriMed-icell-012-sf

Phosphate buffered saline,PH=7.4	Solarbio	P1003-2L
LipofectamineTM 2000 Transfection Reagent	ThermoFisher Scientific	11668019
Triton TM X-100	Solarbio	T8200
CoraLite®594 F-Actin	Proteintech	PF00003
4% paraformaldehyde (PFA)	ThermoFisher	J61899.AP
Bovine Serum Albumin	Gibco	A5256701
BCA protein concentration determination kit	Beyotime	P0010
TRIzol^{TM} Reagent	ThermoFisher Scientific	15596018CN
OPTI-MEM	Gibco	2898884
SKF-96365 hydrochloride(10μM)	MedChemExpress	HY-100001
ML204(0.5μM)	MedChemExpress	HY-12949
Mibefradil dihydrochloride T(2.7μM)	MedChemExpress	HY-15553A
Amlodipine L(30μM)	MedChemExpress	HY-B0317
PD173212 N (10μM)	MedChemExpress	HY-103318
Histamine (10μM)	MedChemExpress	HY-B1204
2-APB(100μM)	MedChemExpress	HY-W009724
Penicillin-Streptomycin	Gibco	15070063
Total Protein Extraction (TPE(TM))	Sangon	C006225
Ca^{2+}-Rhod-2, AM, (5μM,for Live-cell)	YEASEN	40776ES72
Ionomycin calcium (2μM)	MedChemExpress	HY-13434A
Ca++Mg++-ATPase Assay Kit	BIOSS	AK266
Hank's Balanced Salt Solution	ThermoFisher Scientific	14175095
Total Protein Extraction	Sangon	C006225
NucGreen™ Dead 488 ReadyProbes™	ThermoFisher Scientific	S7020
PCR	Novoprotein	E035
**Experimental models: Cell lines**		
C57	Vitalriver	N/A
MSCs	BeijingMaternityHospital	N/A
MC3TE-e1	Peking Union Cell Bank	N/A
C2C12	Peking Union Cell Bank	N/A
iBMSCs	icell	N/A
NIH-3T3	Peking Union Cell Bank	N/A
ESF	Peking Union Cell Bank	N/A
PC12	Peking Union Cell Bank	N/A
HUVEC	Peking Union Cell Bank	N/A
293T	Peking Union Cell Bank	N/A
PX459	Peking Union Cell Bank	N/A
**SiRNASequence (5'-3')**		
si-TRPC4AP-homo<br>GAGGAAACUUACCCAAGAATT	This Study	Gene Pharma
si-TRPM7-homo<br>AACAUCAGACGAACAGAAUUAGUUG	This Study	Gene Pharma

si-TRPM4-homo<br>AUGUGUAACUGAACAUGGCTT	This Study	Gene Pharma
si-TRPV2-homo<br>GCUUCCUUCUGAUCUACUUTT-3	This Study	Gene Pharma
si-ORAI1-homo<br>CGUGCACAAUCUCAACUCGTT	This Study	Gene Pharma
si-ORAI2-homo<br>AACCGUUUGGUUCAAUGAGG	This Study	Gene Pharma
si-STIM1-homo<br>GGCUCUGGAUACAGUGCUCTT	This Study	Gene Pharma
si-TRPC1-homo<br>GCGACAAGGGUGACUAUUATT	This Study	Gene Pharma
si-TRPC4-homo<br>GGUCAGACUUGAACAGGCATT	This Study	Gene Pharma
si-TRPC5-homo<br>UACCAAGGUAUAGAAAUGACUUGGG	This Study	Gene Pharma
**Others**		
96-well cell culture plate	Corning	3548
6-well cell culture plate	Corning	3335
Nunc™ EasYFlask™ Cell Culture Flasks-T25	ThermoFisher Scientific	156367
Nunc™ EasYFlask™ Cell Culture Flasks-T75	ThermoFisher Scientific	156499
Glass bottom cell culture dish (20mm)	NEST	801001
Centrifuge tube-15ml	NEST	601001
Centrifuge tube-50ml-	NEST	602002
